# Price determinants and pricing policies concerning potentially innovative health technologies: a scoping review

**DOI:** 10.1007/s10198-025-01834-y

**Published:** 2025-09-06

**Authors:** Nicolas S. H. Xander, Tom Belleman, Maximilian Salcher-Konrad, Anne Hendrickx, Jeffrey Chen, Anne-Sophie Klein Gebbink, Peter Schneider, Kate Morgan, Oliver Groene, Isabelle Durand-Zaleski, Frederick W. Thielen, Carin A. Uyl-de Groot

**Affiliations:** 1 Erasmus School of Health Policy & Management, Erasmus University Rotterdam, P.O. Box 1738, 3000 DR Rotterdam, The Netherlands; 2 Erasmus Centre for Health Economics Rotterdam, Erasmus University Rotterdam, Rotterdam, The Netherlands; 3 WHO Collaborating Centre for Pharmaceutical Pricing and Reimbursement Policies, Department of Pharmacoeconomics, Austrian National Public Health Institute, Vienna, Austria; 4 Department of Health Policy, London School of Economics and Political Science, London, UK; 5 International Association of Mutual Benefit Societies, Brussels, Belgium; 6 Erasmus Medical Centre, Rotterdam, The Netherlands; 7 Myeloma Patients Europe AISBL, Brussels, Belgium; 8 OptiMedis AG, Hamburg, Germany; 9 Department of Management and Entrepreneurship, Faculty of Management, Economics and Society, University of Witten/Herdecke, Witten, Germany; 10 Unité de Recherche Clinique en Économie de La Santé d’Île-de-France, DRCI, Assistance Publique–Hôpitaux de Paris, Paris, France; 11 Université de Paris Est Créteil, Paris, France; 12 Institute for Medical Technology Assessment, Rotterdam, The Netherlands

**Keywords:** Pharmaceutical pricing, Price determinants, Pricing policies, Patient access, Innovation

## Abstract

**Background:**

Policymakers face challenges in developing pricing policies for potentially innovative healthcare technologies (pIHTs) that balance limited budgets, access, and incentives for innovation. This study aimed to map existing evidence and identify knowledge gaps regarding price determinants and pricing policies for pIHTs and their effect on access and sustainability.

**Methods:**

We conducted a scoping Review of scientific and grey literature in English published between 2014 and September 2023 with pre-specified inclusion and exclusion criteria to identify stakeholder-informed price determinants, pricing policies applied by European Economic Area (EEA) or Organisation for Economic Cooperation and Development (OECD) member states, and their access-related impacts. Literature databases and various stakeholder organisation websites were searched. Further records were included through snowballing and manual addition.

**Results:**

135 Records were included. Stakeholder views on price determinants were available from 15 records and predominantly involved value-based determinants. Pricing policies in EEA/OECD member states are heterogeneous and often feature a mix of policy interventions and implementation methods. External price referencing (EPR), while yielding short-term affordability improvements, is associated with price inequities and launch strategies impairing patient access. Policies combining pricing methods and considering a pIHT’s value have more positive access-related impact but may face feasibility and implementation challenges. Two records mentioned medical device pricing; none featured environmental aspects.

**Conclusion:**

While EPR is commonly applied across Europe, value-informed pricing in connection with health technology assessment is more favoured regarding pIHT access in the literature. Knowledge gaps concern medical device pricing, stakeholder views on price determinants, and the implementation of environmental aspects in pIHT pricing.

**Supplementary Information:**

The online version contains supplementary material available at 10.1007/s10198-025-01834-y.

## Introduction

Health technologies, defined as “medical devices, pharmaceuticals, assistive technologies, techniques, and procedures developed to solve a health problem and improve quality of life” [1], have a substantial impact on healthcare budgets worldwide. The implications of new and potentially innovative health technologies (pIHTs) for the financial sustainability of a country’s health system are primarily determined by their pricing. In this respect, pIHTs are on-patent health technologies whose “innovative” character, especially based on (added) therapeutic benefit [2], must still be assessed at the time of marketing authorisation. As market mechanisms related to generics are inapplicable to on-patent medicinal products (MPs), increasing prices of pIHTs force payers to address difficult questions regarding affordability and appropriate payment mechanisms, and have been pushing the boundaries of “fairness” for many stakeholders [3, 4]. Indeed, inequalities in accessibility and standards of care are exacerbated by high pIHT prices: expensive new pharmaceuticals and medical devices that would be beneficial to many patients are often only available to a selective group of them [5]. There is also evidence that pharmaceutical manufacturers use their discriminating monopoly power to price according to what each market can bear [6]. Moreover, newly authorised pharmaceuticals are often subject to uncertainty regarding real-world clinical outcomes, cost-effectiveness, and budget impact [7]. Therefore, healthcare decision-makers are challenged to find a balance between supporting innovation and ensuring equitable access to beneficial pIHTs, as well as the health system’s financial sustainability [8, 9].

In response to these challenges – which particularly concern on-patent products as opposed to generics – member states of the Organisation for Economic Co-operation and Development (OECD) have introduced pricing policies [10], i.e., “sets of written principles or requirements for managing the prices” [11]. At a fundamental level, these policies are notably classified into (generally) free pricing and price controls. Price controls can take different forms, such as statutory pricing, price negotiations between the decision-maker and the pharmaceutical manufacturer, and public procurement [12–14]. Furthermore, the pricing of pIHTs can be based on different policy interventions and implementation methods. These include, for instance, value-informed pricing (VIP), cost-based pricing (CBP), and reference-based pricing [10–12, 15, 16]. Depending on the applied intervention, the price of pIHTs can be based on different determinants linked to the technology itself. These price determinants are considered by manufacturers, payers, as well as policy- and decision-makers for the calculation of a health technology’s price.

Research on pricing policy interventions has been conducted by the World Health Organization (WHO) [11]. Moreover, research has been performed regarding price determinants on a macro level [17]. However, there has so far been no comprehensive review concerning stakeholder views on price determinants regarding pIHTs, and pricing policies with a specific focus on pIHTs and their impact on patient access and financial sustainability. Such a review would provide a consolidated overview of potential measures to employ price determinants relevant in practice and access-improving policy interventions.

Therefore, the aim of this review was to identify the determinants of prices of pIHTs and to map pricing policies in member states of the European Economic Area (EEA) and the OECD, while also addressing the policies’ benefits and shortcomings according to the literature. To address these objectives, the following research question was formulated: What is the current state regarding price determinants and pricing policies for new health technologies claimed to be innovative? Sub-questions addressed which price determinants are considered by different stakeholders regarding pharmaceuticals and/or medical devices, as well as their calculation and integration for a price. Further, sub-questions involved pricing policies applied in EEA/OECD member states, transparency elements, and their potential impacts on affordability and availability of pIHTs, equity and financial/environmental sustainability, as well as organisational advantages and disadvantages regarding acceptability, resource use, and feasibility.

## Methods

### Protocol and registration

A scoping review protocol containing the objectives, inclusion criteria and methods for this scoping review was created in accordance with the relevant best practice guidance [18]. It was Registered prospectively with the Open Science Framework on 13 October 2023 (https://osf.io/p3gyd) [19].

### Study design

We conducted a scoping review as this method was the most adequate for our research purpose. Our review aimed to map the existing literature concerning price determinants and pricing policies regarding pIHTs in member states of the EEA and/or the OECD, and to identify knowledge gaps within the scope of the review questions. To this end, we followed the guidelines of the Joanna Briggs Institute methodology for scoping reviews [20] and of PRISMA-ScR (PRISMA Extension for Scoping Reviews) [21].

### Search strategy

Scientific publications and grey literature (such as policy papers, institutional reports, and reports by stakeholder organisations) were considered. For scientific publications, three databases were searched (MedLine via Ovid, Embase, Web of Science Core Collection), complemented by a Google Scholar search. Grey literature was searched on the BASE database and 11 websites of stakeholder organisations (see Online Resource 1). Additionally, a Google Advanced Search was conducted to capture Relevant materials published on the internet, with the first 100 hits being considered and pre-screened.

The final search strategies are presented in Online Resource 1.

Results of searches regarding scientific publications were imported to EndNote before de-duplication. The consolidated de-duplicated results were thereafter imported to Zotero v6.0. Results of searches regarding grey literature were imported to Zotero v6.0 and then de-duplicated.

Additionally, references of records deemed relevant for this review were scanned to derive further potential records (snowballing approach).

### Study selection

Publications were included if they focused on price determinants regarding pIHTs. Further, papers on pricing policies applied in practice by member states of the EEA and/or the OECD, as well as their impact on access to pIHTs, and advantages and shortcomings of such policies were considered for inclusion. The focus was on pricing policies applied in individual countries. Further, publications needed to be published in English as well as in the period between 2014 and September 2023.

The initial screening conducted by two reviewers involved an assessment for inclusion based on title and – where available – abstract/executive summary against the inclusion criteria. The screening was AI-aided by ASReview v1.2.1 [22], which features an active-learning mechanism [23].This mechanism is initially trained with a small number of records labelled as relevant or irrelevant by the reviewer. Based on this information, the software shows the reviewer titles and abstracts it considers likely to be relevant, with each reviewer decision (relevant/irrelevant) training the software further [23]. As this aims to show the relevant records early in the screening process, a large number of records labelled as irrelevant in a row indicates that all relevant records may have been found [24]. Therefore, a stopping rule was implemented: the screening ended once 100 records in a row were considered irrelevant. Additionally, a Microsoft Excel® file containing the records’ metadata was created to document the reviewers’ decisions on each screened record’s inclusion.

Thereupon, four reviewers performed a full-text review of the records included after the initial screening and records found through snowballing, and independently decided on their eligibility for the review. Publications in any language other than English, in the form of conference abstracts, opinion papers, as well as comments and replies to previous publications were excluded. Records that did not contain information relevant to the review questions were considered ineligible. For instance, price determinants that are derived from theoretical and model-related considerations and are not based on input from stakeholder groups were disregarded. Likewise, information on pricing policies that were not implemented in EEA/OECD countries or are outdated was not considered. Table 1 contains a detailed overview of the applied inclusion and exclusion criteria. Online Resource 2 shows a list of the exclusion criteria applied in the data extraction form.

**Table 1:** Inclusion and exclusion criteria

**Inclusion criteria**	**Exclusion criteria**
Type of publication: - Scientific paper (published in a peer-reviewed journal) - grey literature study (e.g., report for/by international organisations, academic working paper) - policy paper - magazine article	Type of publication: - conference abstract - comment/reply/correction to previous publication - opinion paper
Date of publication: - published between January 2014 and September 2023 (cut-off date for literature search)	Date of publication: - published before January 2014
Language of publication: - English	Language of publication: - Any other language than English
Content of publication: - Price determinants for branded, on-patent health technologies (pIHTs) based on stakeholder views - Pricing policies regarding pIHTs currently applied in EEA/OECD member states - Transparency as an element of these pricing policies - Impact of pricing policies on patient access (affordability, availability, equity), financial and/or environmental sustainability, or of any other kind - (Dis-)advantages of pricing policies regarding acceptability, resource use, feasibility	Content of publication: - price determinants not explicitly based on stakeholder views (e.g., based on theoretical considerations, econometric models) - pricing policies applied in countries outside the EEA/OECD - outdated or hypothetical/modelled pricing policies - price determinants or pricing policies not applicable to pIHTs - pricing policies dedicated to off-patent health technologies (e.g., generics, biosimilars)

Abbreviations: *EEA* European Economic Area; *pIHT* potentially innovative health technology; *OECD* Organisation forEconomic Co-operation and Development

During the initial screening and the full-text review/data extraction phase, disagreements between reviewers were resolved by consensus.

Additional records were added manually if they were known by the authors to contain relevant information, but not among those included through the described screening and review process.

### Data extraction and synthesis

Data extraction and charting was conducted using an extraction tool set up in Microsoft Excel®. Four reviewers charted data from each eligible article that contained information relevant to at least one of the review questions. Online Resource 3 contains a list of variables included in the data extraction tool.

We extracted the following data points: article metadata; type of health technology and therapeutic area; stakeholder views on price determinants; country of focus regarding pricing policies and applied pricing policies on pIHTs; potential impacts of pricing policies on affordability, availability, equity, financial/environmental sustainability; (dis-)advantages of pricing policies regarding acceptability, resource use, and feasibility.

We summarised the extracted information by each review question and recorded how often different concepts were mentioned. For price determinants considered by stakeholders and their integration in the calculation of a price, we summarised the findings by the respective category (see Online Resource 3). Moreover, for pricing policy interventions and implementation methods, we recorded the countries in which they are applied, as well as information on their potential access-related impacts, as well as organisational (dis-)advantages through qualitative synthesis.

## Results

### Selection and characteristics of sources of evidence

**Fig. 1:**
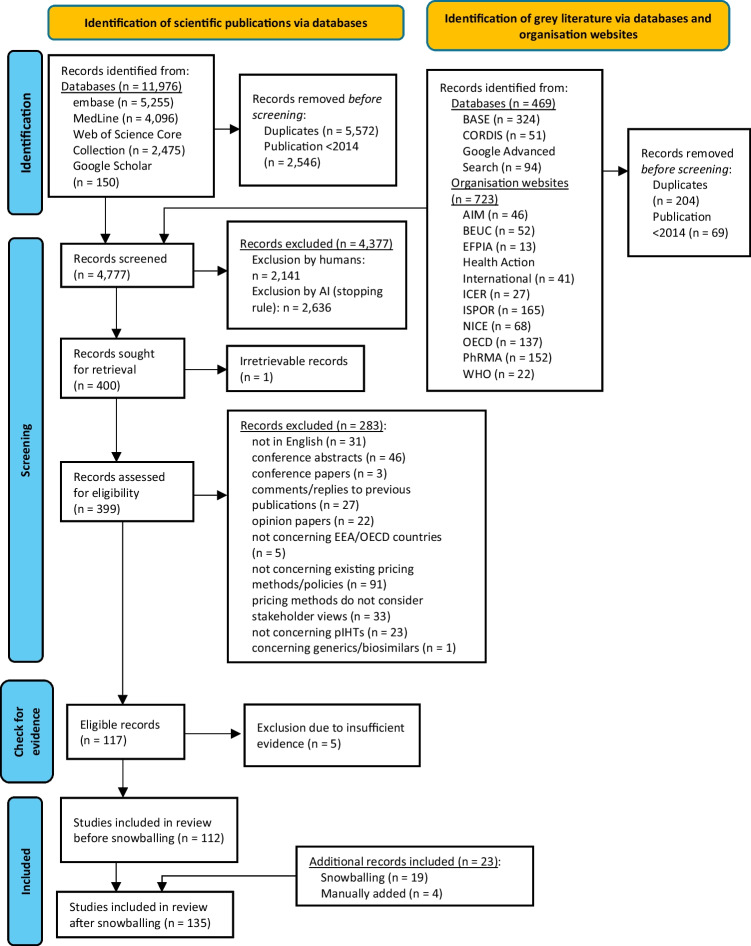
PRISMA flow diagram of literature search and selection criteria. Abbreviations: AIM, Association Internationale de la Mutualité/International Association of Mutual Benefit Societies; BASE, Bielefeld Academic Search Engine; BEUC, Bureau Européen des Unions de Consommateurs/European Consumer Organisation; CORDIS, Community Research and Development Information Service; EEA, European Economic Area; EFPIA, European Federation of Pharmaceutical Industries and Associations; ICER, Institute for Clinical and Economic Review; ISPOR, International Society for Pharmacoeconomics and Outcomes Research; NICE, National Institute for Health and Care Excellence; OECD, Organisation for Economic Co-operation and Development; PhRMA, Pharmaceutical Research and Manufacturers of America; pIHT, potentially innovative health technologies; WHO, World Health Organization

After Removing duplicates and publications from before 2014, 4,777 of 13,168 identified Records were subjected to title-abstract screening. 4,377 records were subsequently excluded based on the applied exclusion criteria (see Online Resource 2). Out of 400 Records qualifying for the full-text review, one could not be retrieved, and 283 were excluded for various reasons (see Fig. 1). Five records were further considered ineligible due to insufficient information as to the review questions (e.g., no further description of an applied pricing policy except for by labelling the pricing policy applied in a relevant country without any further description). Finally, 135 records were selected for data extraction (see Fig. 1).

A total of 104 included Records were scientific articles, of which 94 were peer-reviewed; peer Review status on ten articles was unclear. Moreover, 31 Records were considered grey literature, of which three consisted of policy papers. 58 records were linked to academic funding or first author’s affiliation, 19 to the pharmaceutical industry, 16 to national governmental institutions, four to international and four to non-governmental organisations; two to healthcare payers, two to patient advocacy, and four to healthcare provision. Eight records were associated with consultancies. Finally, six records were funded by a research fund, and ten by a philanthropic institution. For three records, no funding or affiliation information could be retrieved.

MP pricing was discussed in 133 records: information on stakeholder views Regarding price determinants was found in 15 records (see Table 2) [25–39], and 123 records concerned pricing policies applied in EEA/OECD member states (see Tables 3 and 4) [10, 12, 26, 30, 32, 33, 35, 40–155]; five records contained information on both aspects. Medical device pricing policies were described in two records [156, 157].

**Table 2:** Sources of evidence regarding stakeholder views on price determinants

Reference	Publication funding/affiliation of (first) author	Type of technology	Orientation of price determinants^1^	Relevant stakeholder group, description of price determinants	Calculation of price determinants
[25]	Patient advocacy	Medicinal product	Value-based	**Patients**: value defined as access to effective MPs at affordable prices **Industry:** value defined by degree of innovation and potential patient population receiving therapy	NA
			Other determinants	**Industry**: Current and future R&D investment, portfolio investment decisions, present and future competition **Payers (US)**: focus on high level of uncertainty (positive and tangible health outcomes may not be guaranteed)	NA
[26]	Healthcare provision	Medicinal product	Value-based	**General public**: Treatment for unmet needs generally not prioritised (unless not at the expense of further treatment options for those who had available treatments) **Patient groups**: may be primarily concerned with survival, short-term QoL **Physicians**: focus on evidence-based care, value improvements in morbidity and mortality both for current patients and for future generations	NA
[27]	Industry	Medicinal product	Value-based	**Industry (Europe)**: additional efficacy, improved safety, reduction in related healthcare costs, increased productivity and/or other benefits to patient and society	NA
			Reference-based	**Industry (Europe)**: value created by new MP assessed relative to other medical innovations offering similar value; prices of such treatments are analysed in process of establishing a fair price	NA
[28]	Academic	Medicinal product	Cost-based	**Payers/consultants/researchers (various European countries)**: Cost considerations should be integrated with VBP model for OMPs/ATMPs	NA
			Value-based	**Payers/consultants/researchers (various European countries):** Multi-criteria approach or CE evidence (threshold/threshold range over ICER): - added therapeutic value (clinical or patient-reported) - added QoL - comparative safety profile - organisational impact (e.g., oral administration) - patient-reported experience - disease severity - unmet (clinical) need - financial sustainability	NA
			Other determinants	**Payers/consultants/researchers (various European countries):** sustainability (for price negotiations)	NA
[29]	Academic	Medicinal product	Cost-based	**Payers (Europe):** R&D costs (recognising research failures, including public funding, tax refunds; opportunity costs or company take-overs/buy-outs); development costs; production costs; basic profit	R&D costs ≤ €2.5bn; €250m if undisclosed; Development costs weighted by EU market share and adjusted to target patient population Production costs as disclosed, or assumed (CGT: €60,000/application; biologics €150/month; synthesised active substances €50/month; multiplied by 5 for OMPs); Basic profit 8%
			Value-based	**Payers (Europe)**: reward of therapeutic value with innovation bonus Value assessment criteria: QoL improvement, PFS/OS extension, curative effect, unique MP status, indication of MP in life-threatening/chronically incapacitating disease	Innovation bonus 5–40% of total costs
[30]	Industry	Medicinal product (multi-indication)	Value-based	**Payers**: repurposing of same product; WTP may be lower for same added value compared to a new therapeutic product **Industry (Europe)**: improvements of value-added MPs regarding efficacy, safety and/or tolerability; way of administration/ease of use; new therapeutic uses (indication/patient population)	NA
[31]	Consultancy	Medicinal product	Value-based	**General**: uncertainty around any attribute can cause value gap at launch **Payers:** reduction in total cost of care; budgetary certainty; improved disease outcomes; improved health of population; patient and provider satisfaction **Government/regulators:** improved health of population; budgetary certainty; comparative effectiveness; limiting fraud/off-label promotion; ability to use reference pricing **Healthcare providers**: lower treatment costs; improved disease outcomes; increased care coordination; better patient experience **Employers**: wellness, disease prevention; disease management; treatment adherence; worker productivity **Patients/caregivers**: affordable co-payment; individualised MPs; improved disease outcomes; better QoL; easy-to-understand coverage **Industry**: first/best in class; high unmet medical need; lower development/regulatory/reimbursement hurdles; better patient experience; ability to create shareholder value	NA
[32]	Industry	Medicinal product (multi-indication)	Value-based	**Multiple (UK):** relative value of each indication **Industry (UK):** Value of innovation to be reflected in indication-specific prices	Value of any indication as price maximum
[33]	Industry	Medicinal product	Reference-based	**Industry** (**Europe**; on EPR): Use in price setting: preferable to use EPR as indicator in context of broader P&R methodology that takes other factors into account, provides for flexibility in price negotiations; should be limited to in-patient reimbursed MPs to limit distortive effects; Country selection: cluster countries with comparable GDP per capita (PPP-adjusted), healthcare funding systems, IP standards; 5–7 reference countries as optimal number. EPR system should be flexible enough to allow reference basket adjustments in case of crisis situation in a reference country; Frequency of referencing procedure: ideally limited to product launch, after which competitive forces within markets should lead to price/quantity adjustments over time; revisions should be predictable and limited to reasonable intervals (3 years)	NA
[34]	Healthcare payers	Medicinal product	Cost-based	**Payers (Europe)**: R&D costs; production & overhead costs; basic profit	R&D costs: assumed lump sum €250m; if disclosed, maximum €2.5bn; per patient: €20–1200 (high-prevalence disease); ≤ €1m for ultra-rare disease; 10% increase of initial R&D costs for 2nd/3rd indication; Production & overhead costs: €50 for chemical; €150 for biological; multiplied by 5 for OMPs (due to limited production volume)
			Value-based	**Payers (Europe)**: Innovation bonus according to added therapeutic value compared to alternatives (if available) on the market; Criteria: life-threatening/chronically debilitating/rare disease; existence of alternative; curative MP; if not curative: PFS & OS gain, major QoL improvement	Innovation bonus: 5–40%
			Other determinants	**Payers (Europe)**: target population according to disease prevalence; treatment rate	Treatment rate: assumed percentage of target population (based on disease prevalence)
[35]	Research fund	Medicinal product (OMPs/medicinal products for cardiovascular disease)	Value-based	**Payers**: what is the payer able to afford? Short- and long-term budget impact **Patients (US)**: OOP costs (dependent on insurance scheme) **Government**: Short- and long-term budget impact	NA
[36]	Academic	Medicinal product (new oncological treatments)	Value-based	**Healthcare providers**: evidence-based, effective interventions; efficient delivery **Payers/policymakers**: ability to improve health outcomes, QoL improvement; productivity improvement; reduction of total cost of care **Patients:** QoL, symptomatic improvements, lack of side effects, convenience of dosage **Industry**: continued incentives for development of innovative products, positive impact on population health (costs & benefits over time)	NA
[37]	Consultancy	Medicinal product	Cost-based	**Healthcare providers (US)**: cost of development	NA
			Value-based	**Healthcare providers (US)**: survival benefit/efficacy, toxicity, rarity of disease, population burden of disease; consistency of evidence (based on models by ASCO, Memorial Sloan Kettering Cancer Center, National Comprehensive Cancer Network)	NA
[38]	NA	Medicinal product	Cost-based	**Healthcare providers (US)**: research costs	NA
			Value-based	**Healthcare providers (US)**: health benefits, adverse effects, scientific novelty, relative seriousness of disease	NA
			Other determinants	**Healthcare providers (US)**: calculated price to be compared with actual prices paid by insurers and bulk purchasers	NA
[39]	Patient advocacy	Medicinal product (for rare diseases)	Cost-based	**Patient advocacy (Europe)**: R&D costs, approval costs, market entry and commercialisation costs, costs for planned post-marketing research and patient access schemes; (on case-by-case basis) development failures (if relevant to new therapy/disease area), initial investment before repurposing compound; profit margin	20% profit margin
			Value-based	**Patient advocacy (Europe)**: reward for high-risk investments, reward for genuine healthcare innovation; premium for: first MP for disease without treatment, commercialisation in Europe first, MP is developed from R&D of higher productivity with high cost reduction impact on clin development, manufacturing/delivery, unconventional methods, scientific/technological innovations	Agreed determination of value to adjust cost-based price: Multiplication of base price by factor of 10%–100% Premium: 0%–10% extra
			Other determinants	**Patient advocacy (Europe)**: bonus/malus based on: Clinical trials not conducted in Europe, no early access programme via compassionate use	Bonus/malus: 0%–10% (adjustment of cost-based price)

^1^Price determinants are grouped into cost-based, value-based, reference-based and other determinants

Abbreviations: *AIM* Association Internationale de la Mutualité/International Association of Mutual Benefit Societies; *ASCO* American Society of Clinical Oncology; *ATMP* advanced therapy medicinal products; *CE* cost-effectiveness; *CGT* cell and gene therapy; *EFPIA* European Federation of Pharmaceutical Industries and Associations; *EPR* external price referencing; *GDP* gross domestic product; *ICER* incremental cost-effectiveness ratio; *MP* medicinal product; *NA* not applicable/available; *OMP* medicinal products with an orphan designation; *OOP* out of pocket; *OS* overall survival; *PFS* progression-free survival; *PPP* purchasing power parity; *P&R* pricing and reimbursement; *QoL* quality of life; *R&D* research and development; *UK* United Kingdom; *US* United States; *VBP* value-based pricing

**Table 3:** Sources of evidence regarding applied price calculation methods in EEA/OECD member states

Pricing method	References	Application and relevant EEA/OECD member states
Free pricing	[12, 35, 40–71, 73, 74]	*Free price setting by manufacturers throughout lifecycle*: AU (if MP is placed in open market), CO, DE (highly innovative MPs), DK (outpatient MPs; fixed for 14-day periods linked to reporting obligation by manufacturer), MX (for new MPs without comparator), US (federal level) *Free price setting for first period after market launch*: - DE: first 6 (formerly: 12) months after launch for all MPs; - FR: highly innovative MPs/ATMPs with TAU until negotiated price; - IT: first 12 months for some MPs *Free price setting subject to change during reimbursement decision-making process*: HU, SE, UK (under voluntary scheme; responsible pricing required for CDF)
Reference pricing	[12, 40, 42–47, 52–55, 60–63, 65, 66, 68, 70, 71, 75–120 ]	**EPR:** *Primary pricing tool*: AT, BG, CH (equal weight as negotiations), CL, CY, CZ, ES (for MPs without available alternatives on the market), GR, HR, IE (re-alignment of existing prices), KR (if PhEE is waived), IS, LT, LU, LV, MT (two systems for private market and public sector MPs), MX (private sector), NO, PT (pricing precedes reimbursement decision), RO, SK, SI, TR *Supporting pricing tool*: - Informing price negotiations: CA (sets maximum price at federal level; negotiations at provincial level), CH, DE, DK (inpatient MPs), EE, FI, FR (for ASMR I–III; IV for MPs with costs lower than comparator), IE, IT, JP (for price adjustments; also informing IPR), KR (if no waiver for pharmaco-economic evaluation), PL, SI (for prices at particularly low/high level) - Primary method not specified: AU, BE, FI, HU, IL, IS, NL *Position within pricing policy not specified:* CO (for selected therapeutic groups [oncological MPs]; also GDP-based to determine maximum sales price), CR *Used for informal benchmarking:* NZ *Reference basket size*: - EU MS: AT, BE, CZ (with exceptions), FI (plus CH, IS, NO), GR, HU (plus CH, IS, NO), SK - Eurozone: ES - Individually specified number of countries: AU (2), BG (17), CA (6), CH (9), CO (17), CY (9), DE (15), DK (9), EE (3), FR (4), HR (5; 3 primary reference countries), IE (14), IL (7), IS (4), IT (25), JP (4), KR (7), LV (7), LT (27), MT (12; classified into 3 tiers), MX (4), NL (4), NO (9), PL (30), PT (3), RO (12), SI (3), TR (5) - Country of origin: CY (if price not available for reference countries), LT (if price not available for reference countries) LU, TR (if MP is not authorised in the EU) - Not specified: CL, CR *Comparator price*: - Ex-factory price: AT, BE, BG, CZ, ES, FI, HU, KR, LV, MX, PL, RO, SI, SK, TR - Wholesale price: CY, EE, HR, LV (if price in DK is one of the 3 lowest in basket/ex-factory price not available/no wholesale margin indicated by manufacturer) - Retail price: DE, MT (public sector) *Price calculation method*: - Average across reference basket: AT, BE, CH, CY, DK, FI, HR, IE, IL, IS (outpatient MPs), IT, KR, MT (public sector), MX (weighted average based on sales in reference countries in previous quarter), NL - Median across reference basket: CA - Average across lowest reference prices: CZ, GR, LT, NO, SK - Lowest price in reference basket: BG, ES, HU, IS (inpatient MPs), PL, RO (except for MPs for “special needs”), SI, TR - Highest reference price as maximum: EE - Specific rules: CO (maximum price at 25th percentile of reference countries), FR (price must be between lowest and highest reference price), LV (3rd lowest price as maximum; prices in EE and LT must not be exceeded), MT (algorithm for MPs on private market) - Minimum of countries in which MP must be available: AT (12), CZ (3), GR (3), HU (3), NL (2) - Account for exchange rate fluctuations: CZ, EE (EURIPID), ES (EURIPID if no price is available in Eurozone), TR (70% of previous year’s exchange rate to EUR) **IPR:** *Primary pricing tool*: DE (in case of no added therapeutic benefit), ES (for targeted oncology MPs), JP (therapeutically similar “me-too” products) *Supporting pricing tool*: HR, CZ (if EPR and negotiations are not applicable), IT (informing price negotiations), KR *Position within pricing policy not specified*: AU (based on efficacy and safety relative to listed MPs), DK, EE, NZ, SK (for some therapeutic groups) **Price revisions**: - At launch: BE, DE - Periodically: BG (6 months), CH (3 years), CZ (1 year), ES (2 years), FI (5 years), GR (2 years), LV (4x/year), MX (1 year), NL (not specified; full spectrum of MPs), PL (2–2-3–5 years), PT (1 year, full spectrum), RO (1 year), SI (2x/year), SK (2x/year), TR (1 year) - Ad hoc: BG, NL, PL, PT (in specific cases) - Availability in additional reference country: BE, DK, LV - Other: DE (manufacturer’s request, new available evidence), NL (product-specific), SE (manufacturer’s request, involves value re-evaluation)
Cost-based pricing	[55, 90, 102, 121–123]	*Application in price setting*: - AU: for setting Reimbursement rates; profit margin of 30% on manufacturing costs; - GR: for medicinal products exclusively produced domestically; maximum net profit Rate of 8.5%; - JP: for MPs with no comparator on the market; - UK (under statutory scheme)
Value-informed pricing	[12, 40, 42–47, 54–59, 61–63, 66, 70, 72–74, 78, 96, 97, 99–102, 107, 109, 117, 122–129]	*Price setting dependent on outcome of therapeutic value assessment*: CH, CO (relative therapeutic value based on effectiveness and safety), DE, FR (after reimbursement decision), IT, JP (after reimbursement decision), US (State of New York; supplemental rebates to achieve target price for Medicaid) *Subsequent pricing procedure*: - Price negotiations: CH, CO, DE (in case of added therapeutic benefit or no available therapeutic class for MP with no added therapeutic benefit), FR - IPR: DE (in case of no added therapeutic benefit and available therapeutic class) - Price markup: JP (up to 120% for innovative MPs compared to existing comparator) *Price setting dependent on outcome of economic evaluation:* AU (for PBS listing applications; positive evaluation by PBAC required), HU (cost-effectiveness/budget impact is considered by NIHIFM), IE (Decision by HSE on reimbursement price based on price proposed by manufacturer and review by NCPE), KR, PL, SE, UK (manufacturers to price products so that they are cost-effective against applied threshold) *Use of unspecified or other HTA/value assessment for new MPs*: - Systematic use: BE (innovative nature), DK, HU, LV, LT, MT, NL, NO, PT - Used as part of pricing process: AT, BG, CZ, EE, ES, GR, HR, IS, LU, RO, SI, SK, TR *Subsequently established price*: - List price: UK (if cost-effective) - Wholesale price: SE - Not specified: IE *If proposed price is too high:* - Price negotiations: IE, UK - Reimbursement request rejected: SE *Price revisions/adjustments*: - Ad hoc: UK (within PPRS) - Upon request/due to new evidence: UK (individual MPs) - Price adjustments after reimbursement decision: JP
Other pricing policy interventions	[40, 101, 102, 114, 121, 122, 130, 131]	*Tendering for MPs in inpatient sector*: - Predominant tool: DK, LV, IL, IS, IT, KR, MT, NO, PT (online auctions to set maximum price), SE, SI, UK - Additional tool: AT, BE, BG, CA, CH, CY, CZ, DE, EE, ES, FI, FR, GR, HR, HU, IE, LT, LU, NL, PL, RO, SK, TR *Price Maintenance Premium*: JP (for new MPs with higher efficacy; innovativeness, small market size support, paediatric indication support, JP as first market as criteria) *340B Pricing Programme*: US (discounted ceiling prices for eligible healthcare provision entities)
Medical device pricing	[156, 157]	**Diagnostics**: *CBP*: All EU MS, AU, CA, CH, IL, NO, UK, US *Price setting on contractor level*: US **New devices:** *IPR*: KR (lowest price or 90% of highest ceiling price in same function category) *CBP*: KR (for innovative devices; determined by various factors [manufacturing/importation costs, clinical safety, efficacy, economic impact etc.])

Abbreviations: *ASMR* Amélioration du service médical rendu (France); *ATMP* advanced therapy medicinal products; *CBP* cost-based pricing; *CDF* Cancer Drugs Fund (United Kingdom); *EEA* European Economic Area; *EPR* external price referencing; *EU* European Union; *EUR* Euro; *EURIPID* European Integrated Price Information Database; *GDP* gross domestic product; *HSE* Health Service Executive (Ireland); *HTA* health technology assessment; *IPR* internal price referencing; *MP* medicinal product; *MS* member states; *NCPE* National Centre for Pharmacoeconomics (Ireland); *NIHIFM* National Institute of Health Insurance Fund Management (Hungary); *OECD* Organisation for Economic Co-operation and Development; *PBAC* pharmaceutical benefits advisory committee (Australia); *PBS* pharmaceutical benefits scheme (Australia); *PPRS* Pharmaceutical Price Regulation Scheme (England & Wales); *TAU* temporary authorisation for use (France)

Country abbreviations: *AT* Austria; *AU* Australia; *BE* Belgium; *BG* Bulgaria; *CH* Switzerland; *CL* Chile; *CO* Colombia; *CR* Costa Rica; *CY* Cyprus; *CZ* Czechia, *DE* Germany; *DK* Denmark; *EE* Estonia; *ES* Spain; *FI* Finland; *FR* France; *GR* Greece; *HR* Croatia; *HU* Hungary; *IE* Ireland; *IL* Israel; *IS* Iceland; *IT* Italy; *JP* Japan; *KR* South Korea; *LU* Luxembourg; *LT* Lithuania; *LV* Latvia; *MT* Malta; *MX* Mexico; *NL* Netherlands; *NO* Norway; *NZ* New Zealand; *PL* Poland; *PT* Portugal; *RO* Romania; *SE* Sweden; *SI* Slovenia; *SK* Slovakia; *TR* Türkiye; *UK* United Kingdom; *US* United States

**Table 4:** Sources of evidence regarding applied price implementation methods in EEA/OECD member states

Implementation method	References	Way of application and relevant EEA/OECD member states
Price negotiations	[42–45, 47, 52, 54–64, 66, 68–71, 73, 74, 80, 95–98, 104, 107, 109–111, 116, 117, 121, 123, 124, 126, 128, 132–135]	*Negotiation informed by reference pricing:* EE, ES (for innovative MPs of high therapeutic value), IT (for MPs of high therapeutic value), KR, LV, PL *Negotiations based on/informed by added therapeutic benefit assessment*: CA (at provincial level for reimbursement price; price capped at federal maximum price; negotiations can also be conducted with pCPA); CH (equal weight as EPR), CO (relative therapeutic value based on effectiveness and safety), FR (after reimbursement decision; price setting depends on ASMR level, comparator price, expected sales volume, R&D expenses, advertising expenses, indication/patient population), DE (in case of added therapeutic benefit, or no available therapeutic class for MP with no added therapeutic benefit), IT, JP (after reimbursement decision; *inter alia* concerns price markup for innovative MPs), US (State of New York; supplemental rebates to achieve target price for Medicaid) *Negotiations following pharmaco-economic evaluation*: AU, HU, IE (possible in case of negative recommendation by NCPE), SE (reimbursable MPs; optional negotiations between TLV, SALAR and manufacturer), UK *Negotiations informed by other factors*: US (Medicare Part B & D, selected MPs on the market for 7–11 years; therapeutic advance/fulfilment of unmet medical need, R&D costs, prior federal funding considered) *Negotiations as backup*: CZ (MP is not on the market in at least 3 reference countries), TR (products only available domestically) *Non-specific*: BE, CL, CO (public purchases), MX (for MPs in national formulary, including patented antiretrovirals), NL (high-priced MPs), NZ, SI (for extraordinarily high/low prices) *Failure of negotiation:* - Unilateral price setting: FR - Arbitration proceeding: DE - No reimbursement/listing: KR, UK - Taxes and penalties: US *Price revisions*: - At launch: IT - Periodically: CH (3 years), FR (5 years; negotiation of price reduction), JP (2 years; in case of excess sales volumes/sales volume > JPY 15 billion), MX (1 year) - Based on individual agreement: EE, IT - Ad hoc: IE, IT *Negotiations between manufacturer and healthcare providers:* DE (inpatient MPs)
Multi-indication pricing	[26, 30, 32, 41, 125, 136–141 ]	*Single price across indications*: CL, CY, FI, GR, HU, IE, IL, JP, KR, LT, MT, NL (anchored to price of first indication), NO (anchored to price of first indication), PL (anchored to price of first indication), UK *Single weighted average price*: - Weighted by volume: CA, ES - Weighted by value: AT - Weighted by volume and value: AU, BE, DE, FR - Weighting not specified: SE (cost-effectiveness across all indications required) *Price differentials*: - Based on MEAs/negotiated discounts/rebates: BE, DE, EE, ES (at decentralised level), IT - Possible differentiation by brand: DE, ES - Dependent on dosage/pack size: DK - Differentiation per usage: AU (negotiations to arrive at single weighted average price), LV, US *Price reductions due to additional indications*: AT, BE (one-third rule), CL, ES, IT, JP, KR, LV, NL (if price is not considered cost-effective for follow-on indication; subject to negotiations)
Pricing of OMPs	[61, 64, 90, 94, 95, 99, 118, 136, 142–144]	*No specific pricing policy/application of general pricing policy*: BG, CA, CH, DK, EE, FR (expanded EPR basket), HU, IL, IT, KR, LV, PT, RO, SK, TR *Specific policies*: - Maximum ex-factory price must not exceed average EU price: AT - Free pricing: DE (up to sales amount threshold), FI (with justification), SE - Fixed pricing: AU, ES (based on cost-plus system), IE (decision by HSE), JP (costs + 10%), UK - Less restrictive regulations compared to general pricing interventions: GR (related to EPR), KR (skippable price negotiations; pharmaco-economic evaluation at post-marketing stage for cell and gene therapies) - Price revisions: TR (annual, based on sales amounts) *Price negotiations with manufacturer*: BE, NL
Price transparency	[42, 44, 45, 55, 60, 61, 67–69, 71, 81, 83, 91, 92, 96, 112, 119, 131, 138, 145–152, 157]	*Confidentiality of negotiated/net/discounted prices*: AT, AU, BE, CA, DK, ES, FR, HR, IE, IT, NL (especially high-priced products), NZ, NO (inpatient MPs) PL, PT, UK, US *Disclosure to policymakers*: - Ex-factory price: AT, HR - Public R&D funding: FR (disclosed to public domain) - Not specified price: AU, IT, NZ *Public disclosure of prices*: - Ceiling price: BG, US (340B Pricing Programme; disclosure covered entities) - List price: CA, DE (paywalled), RO (updated quarterly), UK, US (advertisements of prescription MPs reimbursed under Medicare & Medicaid) - Wholesale price: DK, IS, UK - Retail price: CO (minimum and maximum sale and selling price, respectively), SE - Officially set price/reimbursement price: AU, SK - Negotiated/net prices: CH (paywalled), DE (paywalled), IS (representative discounted prices), US (state level: Vermont, Maine) - Not specified: CY, MX, NL - Other: CL (pricing data for MPs procured by public sectors), ES (MP expenditure, ex-factory discounts available in community pharmacies), IS (reimbursement amounts), NL (voluntary mechanisms for price data sharing among hospital network), US (actual prices paid by healthcare providers and insurers under Hospital Outpatient Prospective Payment System Policy; notification of imminent price increases > 16% in Oregon, Nevada; justification for price increase in Vermont, applicable to up to 15 MPs incurring substantial state spending and with acquisition cost increase by at least 50% over past five years/15% over past year) - Price reporting no longer required: CA (net prices); NO (inpatient MPs, since 2016) *Medical Devices:* - KR: intransparent pricing

Abbreviations: *ASMR* Amélioration du service médical rendu (France); *EEA* European Economic Area; *EPR* external price referencing; *EU* European Union; *HSE* Health Service Executive (Ireland); *JPY* Japanese yen; *MEA* managed entry agreement; *MP* medicinal product; *NCPE* National Centre for Pharmacoeconomics (Ireland); *OECD* Organisation for Economic Co-operation and Development; *OMP* medicinal product with an orphan designation; *pCPA* pan-Canadian Pharmaceutical Alliance; *R&D* research and development; *SALAR* Swedish Association of Local Authorities and Regions; *TLV* Tandvårds- och läkemedelsförmånsverket (Sweden)

Country abbreviations: *AT* Austria; *AU* Australia; *BE* Belgium; *BG* Bulgaria; *CA* Canada; *CH* Switzerland; *CL* Chile; *CO* Colombia; *CR* Costa Rica; *CY* Cyprus; *CZ* Czechia, *DE* Germany; *DK* Denmark; *EE* Estonia; *ES* Spain; *FI* Finland; *FR* France; *GR* Greece; *HR* Croatia; *HU* Hungary; *IE* Ireland; *IL* Israel; *IS* Iceland; *IT* Italy; *JP* Japan; *KR* South Korea; *LU* Luxembourg; *LT* Lithuania; *LV* Latvia; *MT* Malta; *MX* Mexico; *NL* Netherlands; *NO* Norway; *NZ* New Zealand; *PL* Poland; *PT* Portugal; *RO* Romania; *SE* Sweden; *SI* Slovenia; *SK* Slovakia; *TR* Türkiye; *UK* United Kingdom; *US* United States

### Stakeholder views on price determinants

Views of stakeholders on how piHT prices should be determined were Reported in 15 records [25–39] (see Table 2). Within these, only one record represented a study directly eliciting stakeholder views [28]. In total, 14 records mentioned value-based price determinants [25–32, 34–39]. Multiple stakeholder groups attached importance to the added therapeutic value of a pIHT in terms of efficacy and safety, while taking into account uncertainty in view of limited evidence at launch [25–31, 34, 36–38]. Moreover, a pIHT’s degree of innovation, severity of the targeted disease, unmet (clinical) need, and the impact on financial sustainability are also considered relevant among stakeholders [25, 28, 29, 34–36]. Healthcare professionals were suggested to value increased care coordination, efficiency of delivery, and better patient experience; in this context, payers also considered patient and provider satisfaction as contributors to value [31, 36]. Industry representatives were suggested to emphasise added therapeutic benefit, the degree of innovation, potential patient population size, and high unmet medical need as relevant value-based determinants [25, 27, 30, 31, 36]. On a societal level, Licking & Garfield (2016) argued that employers attach value to disease prevention and management, treatment adherence, and worker productivity [31]. Similarly, the industry was suggested to attribute an MP’s societal value to increased productivity, but also to other benefits to society [27]. The *Fair Price Calculator* by the payer Association Internationale de la Mutualité/International Association of Mutual Benefit Societies (AIM) has proposed an innovation bonus to reflect an MP’s added value [29, 34]. Similarly, as a patient organisation, the European Organisation for Rare Diseases (EURORDIS) called for a similar approach involving a value-related premium that would be added to a cost-related base price [39].

Moreover, six records mentioned cost-based price determinants [28, 29, 34, 37–39]. In these, Healthcare providers, payers, and patient advocates were suggested to consider costs for research and development (R&D), production and overhead, as well as a profit margin relevant in this regard [29, 34, 37, 39]. Jommi et al*.* (2023) mention payers advocating for the integration of cost considerations within a value-based price model for advanced therapy medicinal products (ATMPs) and MPs with an orphan designation (OMPs), respectively [28]. Furthermore, in connection with R&D costs, payers support the recognition of research failures and the inclusion of public funding, tax refunds, and company takeovers or buyouts, respectively [29]. While patient representatives were suggested to agree with the consideration of costs for research failures (on a case-by-case basis), they also consider costs for market entry and commercialisation, as well as for post-marketing research and patient access schemes [39]. From the pharmaceutical industry, no viewpoints on cost-based price determinants were found.

Reference-based price determinants considered by the industry were featured in two records [27, 33]. From their perspective, establishing a “fair price” for a new MP should involve the consideration of prices of other products that have provided similar value [27]. The European Federation of Pharmaceutical Industries and Associations (EFPIA) argued that external price referencing (EPR) should be Restricted to in-patient reimbursement MPs to mitigate its distortive effects. Reference country baskets should be limited to 5–7 countries and adjustable; moreover, price revisions should be predictable and limited to reasonable time intervals (e.g., three years) [33].

Stakeholder views on other price determinants were found in five records [25, 28, 34, 38, 39]. Based on these, the industry was suggested the inclusion of investments into R&D and the product portfolio into determining a price [25], while indicated preferences of payers concerned sustainability, patient population and treatment rate, and uncertainty [28, 34]. Moreover, healthcare providers were suggested to compare calculated prices with net and wholesale prices [38], and patient advocates to consider clinical trial characteristics [39].

Evidence found regarding stakeholder views on the integration of price determinants in price calculations was scarce (*n* = 4) [29, 32, 34, 39]. The relevant records feature stakeholder views from payers, a patient organisation, and the industry; however, other stakeholder groups were not featured (see Table 2). The *AIM Fair Price Calculator* assumes global R&D costs of €250 million per product if undisclosed, or caps them at €2.5 billion if disclosed. Development costs also consider the target patient population based on disease prevalence and a 50% treatment rate [29, 34]. Production costs are included as disclosed, otherwise assumed at specific fixed amounts depending on compound type (with a multiplier applied to OMPs), and for cell and gene therapies [29, 34]. The calculator applies a basic profit margin of 8% and a value-based innovation bonus of 5–40% [29, 34]. EURORDIS (2018) Recommended a similar approach with a cost-based baseline price compounded by a 20% profit margin; this approach also involves a value-based bonus of 10–100% of the base price and price premiums encouraging R&D investment in areas of particular importance [39]. The AIM *Fair Price Calculator* calculates a pIHT’s price for each new indication, applying a unique weighted average [29, 34]. Furthermore, for MPs used in multiple indications, Neri et al. (2018) suggested that multi-indication pricing should reflect the relative value of each indication; however, the price should not exceed the value of any indication [32].

Table 2 provides a detailed overview of the relevant records as well as price determinants and their integration in calculations in view of the respective stakeholder groups.

### Pricing policies applied in EEA/OECD member states

The included records contained information on applied pricing policies for all EEA/OECD member states except Liechtenstein. Information on pricing policies applied in these countries was extracted from included documents and relates to their time of publication.

Based on the data extraction, a further distinction between quantitative policy interventions – compartmentalised into free pricing, reference-based pricing, CBP, VIP, and other interventions – and pricing implementation methods (classified into price negotiations, multi-indication pricing, OMP pricing, and price transparency) was considered appropriate. This is structurally reflected in the presentation of the relevant results.

Generally, MP-related pricing policies were found to refer to ex-factory or list prices (proposed by the manufacturer). However, in several countries, the set price concerns the pharmacy purchasing (or wholesale) price, or the retail price charged by pharmacies to payers [40, 158, 159]. This contrasts the net price after discounts and rebates, denoting the amount actually received by manufacturer, wholesale, and retail. Furthermore, many countries were found to apply several interrelated policy interventions which could not be isolated from one another (e.g., price negotiations informed by price referencing or value assessment). To illustrate how individual pricing policies are applied in practice and how the decision-making process of the pricing of MPs is timed in relation to their reimbursement, three boxes summarising the policies applied in selected countries were included. These concern pre-reimbursement pricing (Bulgaria; Box 1), peri-reimbursement pricing (Sweden; Box 2), and post-reimbursement pricing (France; Box 3).

Box 1: Bulgaria as an example of pre-reimbursement pricing

**Table:** 

Pre-reimbursement pricing: Bulgaria
EPR as primary pricing tool for Reimbursable and prescription MPs. Maximum price set through EPR is lowest ex-factory price among all 17 countries in reference basket. Reference countries have similar GDP/economic development and/or are neighbouring countries. In case of unavailable price information in a reference country, an alternative reference country is selected.
HTA is used as part of the pricing process; however, included literature does not specify its application in detail.
Procedure: Price approval by NCPRMP; 60 days to approve set price and list MP in PDL (reimbursement decision succeeds pricing).
Price revisions: every 6 months for reimbursed MPs.

Abbreviations: *EPR* external price referencing; *GDP* gross domestic product; *HTA* health technology assessment; *MP* medicinal product; *NCPRMP* National Council on Prices and Reimbursement of Medicinal Products (Bulgaria); *PDL* positive drugs list

Box 2: Sweden as an example of simultaneous pricing and reimbursement procedure

**Table:** 

Simultaneous pricing and reimbursement: Sweden
Manufacturer proposes price in request for reimbursement of a new MP (for outpatient sector).
Subsequently: cost-effectiveness analysis; pricing and reimbursement decision is based on societal perspective, cost-effectivness threshold value, and marginal decreasing utility of treatments (variable benefit based on indication/severity of disease). No reimbursement if proposed price is too high and criteria for positive decision are not met.
Price revisions: TLV can decide on price increase/decrease upon initiative of manufacturer. Further, it can review pricing and reimbursement status to check if MP still provides enough value for the indication(s) it is used for.
MPs used for multiple indications: Weighted average price; must be cost-effective across all indications.

Abbreviations: *MP* medicinal product; *TLV* Tandvårds- och läkemedelsförmånsverket

Box 3: France as an example for the application of post-reimbursement price setting

**Table:** 

Post-reimbursement pricing: France
Reimbursement: precedes pricing procedure; decision takes into account gravity of health problem, efficacy, and public health impact; tiered coverage according to SMR level.
Value-informed pricing following reimbursement decision based on outcome of therapeutic value assessment (ASMR); price setting (list price) through negotiations between manufacturer and CEPS: - ASMR I–III: price is informed by EPR; - ASMR IV: costs must not exceed comparator costs; - ASMR V: price 5–10% lower than comparator; price of nearest comparable drug must not be exceeded.
Further factors considered in negotiations: price of related MPs/comparators, expected sales volume, R&D expenses, advertising expenses, indication/patient population.
If negotiations fail: unilateral price setting by CEPS, or market exit.
Price revisions: every 5 years, through re-negotiation of a lower price.
For highly innovative MPs/ATMPs with TAU: free pricing by manufacturers upon EMA market authorisation; value-informed price negotiations. Difference between freely set price and negotiated price must be repaid.
MPs used for multiple indications: Separate therapeutic value assessments for each indication; set price represents the value across indications weighted by the expected volume (weighted average pricing). Price may be revised in case of disparity between expected and actual volume weight.
OMPs: Same price setting procedure and criteria apply. If EPR is applied: expanded reference country basket.

Abbreviations: *ASMR* Amélioration du service rendu; *ATMP* advanced therapy medical product; *CEPS* Comité Economique des Produits de Santé (France); *EMA* European Medicines Agency; *EPR* external price referencing; *MP* medicinal product; *OMP* medicinal product with an orphan designation; *SMR* service médical rendu; *TAU* temporary authorisation for use

#### Quantitative policy interventions for medicinal products

Several EEA/OECD member states employ *free pricing* of reimbursable MPs by the manufacturer either as a primary or a partial policy intervention [12, 35, 40–71, 73, 74]. Free pricing of on-patent MPs is allowed in the United States (US) and Denmark [35, 42–53]. However, the latter imposes bi-weekly price reporting obligations by manufacturers and EPR-informed price ceilings for inpatient MPs [71]. Germany and Italy allow temporary free pricing for a specified period after market launch [12, 42–47, 52, 54–61, 73]. In Hungary, manufacturers can freely set a price for a new MPs but may be forced to reduce it during the reimbursement procedure [62, 63]. Moreover, free pricing is closely linked to HTA-informed price limits in countries where reimbursement decisions are informed by a MP’s cost-effectiveness: price setting liberties are only restricted if the respective MP is not cost-effective compared to the applicable comparator [41–43, 52, 74]. Table 3 contains a more detailed overview of the application of free pricing across EEA/OECD member states.

*Reference-based pricing* is a frequently applied policy intervention, with almost all EEA/OECD member states except for the US, the United Kingdom (UK), and Sweden applying it as part of their MP-related pricing policies [12, 40, 42–47, 52–55, 60–63, 65, 66, 68, 70, 71, 75–120]. EPR is used as a primary or supportive policy intervention of newly authorised MPs (see Table 3). As a primary tool, it is applied in small to medium-sized MP-related markets (e.g., Iceland, Luxembourg, Malta, Cyprus, Norway) and countries in Southern, Central, or Eastern Europe, including Türkiye [52, 62, 76–94]. Outside of Europe, South Korea considered EPR for new MPs not subject to a pharmaco-economic analysis [95]. As a supporting tool in connection with other pricing policy interventions, various EEA/OECD countries apply EPR to inform price negotiations with manufacturers (e.g., Belgium, Estonia, France, Germany, Italy, Poland, Spain, and South Korea) which, in some countries, follow VIP-based approaches [42–44, 52, 54, 55, 64, 76–80, 82, 96–99, 118], to limit freely set prices for in-patient MPs (Denmark) [52, 77], or, respectively, for MPs considered innovative or with significant added therapeutic value (Canada, Japan) [42, 43, 65, 77, 78, 100–102, 119, 120]. For some countries, no included record specified how EPR as a supportive tool informs the primary policy intervention, nor what that primary intervention is (see Table 3) [52, 62, 63, 75–79, 82, 103, 104]. Heterogeneity was found across countries regarding the size of the respective reference country baskets, the referenced type of price (ex-factory price, wholesale price, retail price), the applied reference price calculation method, and the implementation of price revisions (see Table 3).

Compared to EPR, the application of internal price referencing (IPR) to pIHTs is limited. There is heterogeneity across countries regarding the relevant MPs – e.g., products without added therapeutic benefit (Germany) or so-called “me-too” products (Japan) – and the use of IPR as a policy intervention, e.g., as a tool to inform price negotiations (Italy, South Korea), to determine the reimbursement price cap (New Zealand), or as a weighting factor of the reimbursement price (Switzerland) (see Table 3) [43–45, 53–57, 59–61, 77, 79, 95, 97, 101, 102, 107, 108].

As a pricing policy intervention, *CBP* is rarely applied (see Table 3) [55, 90, 102, 121–123]. Japan uses it to price MPs without any comparator [102, 121, 122] and has implemented a repricing system under which prices are revised if annual sales exceed specific thresholds [123]. The UK applies CBP to MPs under the Statutory Scheme – as opposed to the voluntary scheme, which involves VIP – by considering development costs and profit margin [55]. Greece applies CBP to fully domestically produced MPs [90].

Based on the included literature [12, 40, 42–47, 54–59, 61–63, 66, 70, 72, 73, 78, 96, 97, 99–102, 107, 109, 117, 122–129], the application of *VIP* across EEA/OECD member states can be classified into two main categories. The first concerns an MP’s therapeutic value assessment against the applicable comparator. Such assessment is limited to clinical criteria and does not consider economic aspects such as cost-effectiveness. In countries applying this approach (see Table 3), the assessment outcome informs further policy interventions (e.g., IPR in Germany for MPs without added therapeutic benefit) and implementation methods, such as price negotiations, e.g., in Germany (for MPs with added therapeutic benefit), France, Italy, Switzerland, and the State of New York (for high-cost MPs), or price premiums (Japan) [42–47, 55, 57–61, 66, 73, 96, 109, 110, 122–126].

The second VIP category involves the employment of a pharmaco-economic evaluation to estimate a newly authorised MP’s cost-effectiveness compared to the applicable comparator based on a given price. Several countries – Hungary, Ireland, Poland, Sweden (see Box 2), Australia under the Pharmaceutical Benefits Scheme, South Korea, and the UK under the Voluntary Scheme – apply such evaluations to inform decision-making on reimbursement as well as price setting through the use of cost-effectiveness thresholds, setting *ex ante* limits to the reimbursement price in view of the MP’s added benefit (see Table 3) [12, 42–44, 52, 55, 62, 63, 74, 97–100, 107, 127–129]. The subsequent pricing procedure differs per country (see Table 3). Multiple other EEA member states such as Latvia, Lithuania, the Netherlands, and Norway employ HTA and/or value assessments for pIHTs either systematically or as part of the pricing process (see Table 3) [40]. However, no information was found on the detailed role of such assessments within the pricing procedure in those countries.

Furthermore, we found pricing policy interventions concerning MPs in the included literature that either contain unique features, or did not fall under the previously described interventions [40, 101, 102, 122, 123, 130, 131]. These involve tendering applied to inpatient MPs [40], the federal 340B Pricing Programme in the US which has established a ceiling price for outpatient MPs based on Medicaid rebates [130, 131], and the Price Maintenance Premium in Japan which applies a price premium to an MP over the comparator price based on value-, indication-, and market-based criteria [101, 122, 123].

Table 3 provides a more detailed overview regarding the described applied pricing policy interventions.

#### Quantitative policy interventions for medical devices

According to the included literature, CBP is suggested to be generally applied to medical devices [156]. In the US, pricing decisions are mostly determined by the market and by local contractors [156]. Conversely, South Korea involves multiple clinical and economic factors in the pricing of innovative medical devices dissimilar from listed products in the same functional group [157] (see Table 3).

#### Pricing implementation methods

Based on the included literature, multiple EEA/OECD member states set MP prices through *negotiations* with the manufacturer [42–45, 47, 52, 54–64, 66, 68–71, 73, 74, 80, 95–98, 104, 107, 109–111, 116, 117, 121, 123, 124, 126, 128, 132–135]. These are often informed by an assessment on an MP on its added therapeutic benefit or cost-effectiveness [42–45, 47, 52, 54–61, 63–66, 107, 120, 129, 132]. Further, some countries used reference prices obtained through EPR/IPR as a negotiation basis [54, 79, 80, 109, 111]. Additionally, negotiations can be limited to pre-selected (US) or high-priced MPs (the Netherlands, Slovenia) or employed as a backup method in case of specific policy interventions not being applicable to the respective MP [52, 78, 80, 126, 133–135]. Consequences of negotiation failure can take several forms, namely unilateral or arbitration-based price setting, denial of reimbursement/listing, and sales-volume-based penalties against the manufacturer [42–45, 54–61, 66, 96, 133–135]. Table 4 contains a detailed overview of the application of price negotiations.

For *MPs approved for multiple indications*, indication-related price implementation was found to be heterogeneous across EEA/OECD member states [26, 30, 32, 41, 125, 136–141]. Firstly, several countries set a single price across all indications, with some basing it on the price for the first approved indication (e.g., the Netherlands, Norway, Poland), on a weighted average with the weight being determined by volume and/or value (e.g., Austria, Belgium, Germany, France, Spain, Canada), or a price cost-effective at all indications (Sweden, UK) [30, 32, 125, 136–140]. Secondly, some countries apply price differentials based on differences in brand, dosage or usage, or through nationally or regionally negotiated agreements [32, 125, 136–138, 140, 141]. Thirdly, multiple countries impose price reduction upon an MP receiving marketing authorisation for follow-on indications [125]. A more detailed overview is provided in Table 4.

The pricing of *OMPs* was found to depend on the existence of implementation methods specific to such products [61, 64, 90, 94, 95, 99, 118, 136, 142–144]. Most EEA/OECD member states do not apply price implementation methods specific to OMPs. Rather, either the general policy interventions and implementation methods apply, or manufacturers can price such products freely. Conversely, countries applying specific pricing regulations for OMPs do so in a heterogeneous manner (see Table 4). Regarding cell (and gene) therapies, France and South Korea apply modified regulations that facilitate faster market entry [64, 99, 118].

Finally, the included literature suggested differing levels of *price transparency* [42, 44, 45, 55, 60, 61, 67, 68, 71, 74, 81, 83, 91, 92, 96, 112, 119, 131, 138, 145–152, 157]. Generally, while manufacturers often disclose ex-factory/list prices, negotiated net prices remain confidential and therefore inaccessible to other stakeholders and the public in most countries (see Table 4). On a national level, some countries make wholesale and/or retail prices publicly available (e.g., Bulgaria, Croatia, Cyprus, Denmark, Iceland, Netherlands, Romania, Sweden) [67, 74, 81, 83, 91, 145]. While Germany, Iceland, and Switzerland provide access to net price information of MPs, such information is only partial and/or behind a paywall [55, 60, 67, 145]. Finally, several states in the US (Vermont, Maine) obligate manufacturers to disclose net prices [146, 152]. Medical device pricing in South Korea was considered untransparent [157]. Table 4 contains detailed information regarding the implementation of MP price transparency.

### Access-related impacts and organisational advantages and disadvantages of applied pricing policies

Information on *access-related impacts and/or organisational (dis-)advantages of pricing policies* applied in EEA/OECD member states in general and with Respect to specific countries, respectively was provided in 71 records. An overview is provided in Tables 5 and 6; Online Resources 4 and 5 contain more detailed impact-related information. No data was found regarding the impact of applied pricing policies on environmental sustainability.

**Table 5:** Sources of evidence on access-related impact and organisational (dis-)advantages of applied quantitative pricing policy interventions in EEA/OECD member states

Policy intervention	References	Description of impact^1^	Description of (dis-)advantages^2^
Free pricing	[35, 43–45, 48, 49, 65, 71]	**Affordability:** - Strong competition through frequent price changes, thus lower MP prices (DK); - Detrimental effect of high and rising launch prices, price increases on affordability for payers and patients (US) **Availability:** - Rapid access and earlier availability (DE, US); - TAU allows market launch of highly innovative MPs before price negotiation at freely chosen price (FR) - States with higher MP costs for Medicaid: services cuts, increased eligibility requirements (US) **Sustainability:** NA **Equity:** - Uninsured patients cannot afford expensive MPs (US) **Other impact:** - Divergent growth of US MP prices implies increasing strain on reimbursement negotiations in other countries if manufacturers aim to maintain income-related differentials (US); - Increased deductibles and co-payments/coinsurance may reduce adherence to effective medications	NA
Reference-based pricing	[10, 12, 33, 52, 75, 76, 78–86, 91, 93, 96, 101, 103, 105, 108, 112, 113, 115, 120, 153–155]	**Affordability:** - EPR: can lead to substantial short-term savings for public payers (general); introduction and application has led to lower MP prices (BG, CO, GR [9.5% decrease in 2010], NL, RO, SK [expected price reductions of €75 million by 2012], TR) - EPR: affordability problems due to reduced manufacturers’ willingness to price to market; - EPR: referencing official list prices instead of adjusting for discounts/rebates leads to risk of payers (substantially) overpaying - EPR: Pricing-related inequities undermine affordability (low relative price levels in countries with high absolute price levels and vice versa) – might also partially be explained by parallel trade; - EPR: price instability as cross-border spill-over effect **Availability:** - EPR: launch sequence strategies by manufacturers prioritize accelerated market entry in countries without direct price controls (DE, IE, UK) and lead to launch reluctance/access delays regarding relatively low-income countries and countries with lower prices that would affect other countries in Europe (BE, BG, PT, TR); - EPR: MP shortages due to parallel trade and market withdrawals (BG [200 products withdrawn since 2012], RO [> 1175 products having disappeared from the market]); - EPR: price revisions in a country may trigger circular price revision sequences, further contributing to strategic launching; - EPR: increasing size of reference country baskets may lead to low-WTP countries not being served (general), disregard for new prices and exemption lobbying (SK); - IPR: manufacturers wary to accept a low price may lead to delayed launch of new products **Sustainability:** - EPR/IPR: can contribute to short-term cost containment (general, British Columbia [CA – CA$161 million saved in first six years after IPR implementation], SK, TR [achieved savings of around US$1 billion), but ineffective in the long run (GR); savings lessened due to increased reimbursement and number of patients entitled to reimbursement (BG); - EPR: regular price revisions can lead to greater short-term cost containment due to lower price levels; **Equity:** - EPR: relatively higher MP prices in countries with lower absolute price levels **Other impacts:** - EPR: real prices often remain unknown due to confidential discounts (especially for high-priced MPs); - EPR: spill-over effects (also due to parallel trade) likely to lead to access issues, limited cost savings, and negative impact on R&D investments; - EPR: (downward) price convergence reduces industry revenue (general, CH [reduction by €430 million) and potentially discourages incremental innovation (reduced revenue leads to reduced R&D investment potential); - EPR: limits viability of other price regulation methods, especially of VIP; also leads to path dependence (observed price levels are influenced by EPR rules in individual countries and ignore other market aspects such as health needs, income, healthcare costs)	**Acceptability:** - EPR: widely accepted, commonly applied cost-containment tool and popular starting point for price negotiations (general); price calculations for new MPs considered acceptable by key stakeholder groups (HR); - EPR: indication of “benchmark” prices considered major benefit by several policy-makers; - Resulting inequity between high- and low-GDP countries considered unacceptable **Resource use:** - EPR: technically and administratively complex, requires large amounts of data, cost- and time-intensive application **Feasibility:** - EPR: no investments in HTA/pharmaco-economics required, as opposed to VIP; - EPR: lack of available price information, price heterogeneity, confidentiality of discounts/rebates, exchange rate volatility, price reductions not automatically being translated complicate implementation; - EPR: inefficient approach for price reduction when used in isolation
Cost-based pricing	[101, 121, 122]	**Affordability:** - Helps patient populations with rare diseases be protected from monopoly prices **Availability:** - Empirical effect uncertain **Sustainability:** NA **Equity:** NA **Other impacts:** - Costs of failed R&D efforts might not be recovered, which could adversely influence investments	**Acceptability:** NA **Resource use:** NA **Feasibility:** - Calculation of indirect expenses is subject to manufacturer arbitrariness
Value-informed pricing	[12, 42–45, 57, 58, 60, 72, 73, 96, 97, 103, 111, 128]	**Affordability:** - Incremental treatment costs between launch and price negotiation decreased by 24.5% due to AMNOG (DE); added value approach saves money (FR); - Taking into account high- and low-value indications is perceived as the best way to ensure affordability of novel therapies (DE); - Given allocated healthcare budget, potential affordability issues can occur despite an MP’s cost-effectiveness (UK) **Availability:** - Price negotiations after market entry should not delay market access; no market withdrawals observed for MPs with added benefit for at least one patient group (DE); - 90-day launch deadline following positive NICE recommendation incentivises threshold-compliant pricing and thus faster availability due to automatic access (UK) **Sustainability:** - Better fund allocation; MP spending growth has been stopped without slowing access to innovative MPs (FR) - accumulated savings of €14 billion for health insurance funds over ten years, but uncertainty of effect on prescription MP expenditure (DE); - lower MP prices were achieved (KR) **Equity:** - Savings from price decreases of older MPs facilitate financing of innovative and expensive new MPs (FR) **Other impacts:** - Potential incentive for development of products that generate more added value; - Too low price in relation to value could discourage development of new MPs in the long run due to lack of reward for innovation; - Opportunities for manufacturers to “game the system” related to choice of comparator and cost-effectiveness threshold-based price proposals; - Closer alignment of prices with clinical benefits (DE)	**Acceptability:** - Logical and fair policy to promote access and reward useful innovation; - Early public opinion supportive of AMNOG (DE); manufacturers accepting of lower prices if reimbursement is conditional on added therapeutic benefit over existing products priced at same/lower amount (FR); - WTP-corresponding price levels may lead to uneven distributions of a product’s benefit surplus between payers and manufacturers, and volatility of R&D returns (UK) **Resource use:** - Evidence-based MP pricing allows pharmacists to focus more on clinical and less on economic activities (general); - Value assessments are resource- and time-intensive (general) **Feasibility:** - Difficult to implement, especially in therapeutic areas with no alternative treatment and patients suffering from severe life-threatening/debilitating diseases; would also require revisit of coverage and price negotiation rules in countries where such system is not applied (general, US); - Perceptions of value may differ across stakeholder groups (general); - Value-informed price regulation is insufficient to control spending (FR)
Other policy interventions	[101, 102, 122, 123, 130, 131]	**Affordability:** - 340B Pricing Program reduces acquisition costs (US) **Availability:** - 340B Pricing Program helped promote access increase to high-cost oncology services (US) **Sustainability:** - PMP may increase healthcare spending if premiums are overly generous (JP) **Equity:** - 340B Pricing Program suggested to have increased access to oncology services in rural communities **Other impacts:** - PMP: promotes innovation; conversely, sales-related price revisions may discourage innovation (JP); - 340B Pricing Program: lack of specificity in patient eligibility guidelines enables too broad/narrow interpretation	**Acceptability:** - PMP can be considered a reasonable approach to evaluate value of new MPs (JP) **Resource use:** NA **Feasibility:** - Price calculation expected to be difficult (JP)

^1^Types of impacts of pricing policies are grouped into affordability, availability, equity, sustainability, and other

^2^Advantages/disadvantages of pricing policies are grouped into acceptability, resource use, and feasibility

Abbreviations: *AMNOG* Arzneimittelmarktneuordnungsgesetz (German Medicines Market Reorganisation Act); *CA$* Canadian dollar; *EPR* external reference pricing; *GDP* gross domestic product; *IPR* internal reference pricing; *MP* medicinal product; *NA* not available/not applicable; *NICE* National Institute for Health and Care Excellence (England & Wales); *PMP* Price Maintenance Premium (Japan); *R&D* research and development; *TAU* temporary authorization for use (France); *US$* United States dollar; *VIP* value-informed pricing

Country abbreviations: *BE* Belgium; *BG* Bulgaria; *CA* Canada; *CH* Switzerland; *CO* Colombia; *DE* Germany; *EE* Estonia; *ES* Spain; *FR* France; *GR* Greece; *HR* Croatia; *HU* Hungary; *IT* Italy; *JP* Japan; *KR* South Korea; *LV* Latvia; *MX* Mexico; *NL* Netherlands; *NZ* New Zealand; *RO* Romania; *SE* Sweden; *SK* Slovakia; *TR* Türkiye; *UK* United Kingdom; *US* United States

**Table 6:** Sources of evidence on access-related impact and organisational (dis-)advantages of applied price implementation methods

Implementation method	References	Description of impact^1^	Description of (dis-)advantages^2^
Price negotiations	[44, 45, 56, 57, 66, 73, 97, 116, 121, 132–135]	**Affordability:** - Policy sets limits on what purchasers pay for an MP; - Low MP prices possible due to monopsony and bargaining powers in negotiations; insufficient empirical evidence for effectiveness (MX); - May improve through Medicare MP price negotiation (US) **Availability:** - After market price negotiation should not delay patient access; - Availability depends on negotiation/arbitration outcomes and manufacturer decision: 29/148 MPs withdrawn from market (2011–2017) following negotiation/arbitration (DE); - Negotiation with monopsonistic purchasers and production at lower quantities compared to competitive market impairs access to goods and services **Sustainability:** - Potential decrease in MP expenditures through pCPA-led price negotiation (CA); - Estimated savings of US$98.5 billion over ten years through Medicare negotiation; 5.4% spending reduction on Medicare Part B and D **Equity**: NA **Other impacts:** - Manufacturer risk losing an entire national market unless they negotiate; - Need for more transparency and consistency confirmed through MP price negotiation (KR); - Potential incentive to increase MP prices and introduce alternative versions to evade Medicare price negotiation (US)	**Acceptability:** - Arbitration constitutes politically legitimate means for price setting in case of negotiation failure (DE) **Resource use:** NA **Feasibility:** NA
Multi-indication pricing	[26, 32, 41, 111, 136–138, 141]	**Affordability:** - Potential decrease on payers’ expenditure, but increased price in high value indication can mitigate this effect - With each new indication, MPs’ list prices were significantly reduced (DE, FR) **Availability:** - MPs for low-value indications may not be launched, even in countries applying weighted average pricing or differential discounts; - Fixed price across indications carries risk of limited access if cost-effective indication is not reimbursed (SE) **Sustainability:** - Potential net increase in spending due to access to MP that would otherwise not be paid at “asking price” **Equity:** - Patients with higher-value indication should not face the same cost sharing as patients receiving low-value indication MPs; administrative burden makes this alignment more difficult **Other impacts:** - Little incentive to launch MP for indication with smaller patient populations due to potential adverse effects on list price and thus profits; - Potentially improved alignment of individual product access, MP value and price; - Potential optimisation of R&D incentives and increase of competition; - Potential reluctance of payers to acknowledge added clinical benefit; - Manufacturer can capture all the economic surplus for each indication	**Acceptability:** - Potential opposition of key stakeholders to indication-specific pricing; - ATC-based pricing is preferred over a complex multi-indication pricing system (ES); - Administrative complexity and large transactional burden from negotiation discourages payers (UK) **Resource use:** - Increase in administrative costs associated with indication identification, differentiation of value and payment process; - Data systems for indication-specific pricing models may be complex and difficult-to-use **Feasibility:** - Adequate infrastructure required to obtain necessary information to determine the value of MP and suitable institutional framework for evaluation; - Weighted-average pricing is complicated (considering competition and its impact on sales volume); - Legal/regulatory constraints might hinder implementation of multiple prices - The pricing system embeds different values of an MP across indications (DE)
Price transparency	[55, 145, 147, 148, 150 ]	**Affordability:** - Sharing negotiated prices of oncological MPs was associated with lower prices over time; - Hospital price transparency: Having price information may enable patients to be in control and lower the costs they faced (NY/US); - Information sharing of among hospitals on MP prices led to savings on brand that was previously overpaid (US) **Availability:** NA **Sustainability:** - Inconclusive evidence on whether MP pricing transparency reduces MP spending due to various factors involved **Equity:** NA **Other impacts:** - Manufacturer: confidential negotiations allow larger discounts and improve payer’s ability to negotiate lower prices - Possible positive collateral effect of net price disclosure; other countries can reference net prices on basis of an MP’s clinical value (DE)	**Acceptability:** NA **Resource use:** NA **Feasibility:** - Transparency alone is insufficient to encourage patients to price-shop; evidence suggests only modest changes in patient behaviour - Combining transparency with targeted consumer incentives can lead to widespread price-shopping - In combination with tangible financial incentive, MP spending can be reduced

^1^Types of impacts of pricing policies are grouped into affordability, availability, equity, sustainability, and other

^2^Advantages/disadvantages of pricing policies are grouped into acceptability, resource use, and feasibility

Abbreviations: *ATC* anatomical therapeutic chemical; *MEA* managed entry agreement; *MP* medicinal product; *NA* not available/not applicable; *OOP* out of pocket; *R&D* research and development; *US$* US Dollar

Country abbreviations: *CA* Canada; *CH* Switzerland; *DE* Germany; *ES* Spain; *FR* France; *IT* Italy; *KR* South Korea; *MX* Mexico; *NY* New York (state); *SE* Sweden; *SK* Slovakia; *UK* United Kingdom; *US* United States

#### Quantitative policy interventions

High prices of freely priced MPs were suggested to incur detrimental affordability issues and enable inflation-exceeding price increases (US) if no monitoring, like in Denmark, is in place [35, 43, 48, 71]. Consequently, divergent price growth in the US and Europe were argued to increase the strain on pricing and reimbursement negotiations outside the US if manufacturers seek to maintain income-related differentials relative to US prices [49]. By contrast, free pricing was associated with early availability compared to other markets [43, 45, 65]. Indeed, Germany’s approach of a limited free pricing period upon launch was found to allow quick patient access to newly authorised MPs and facilitate one of the highest availability levels across Europe [45, 57].

Based on the included literature regarding the impacts and organisational (dis-)advantages of *reference-based pricing* [10, 12, 33, 52, 75, 76, 78–86, 91–93, 96, 101, 103, 105, 108, 112, 113, 115, 120, 153–155], EPR was suggested to yield short-term cost containment and savings for payers. In some Eastern and Southeastern European countries, the introduction of EPR was associated with subsequent (absolute) price reductions and healthcare savings (see Table 5) [82, 83, 85, 91, 103, 114]. However, these were suggested not to persist in the long term [52, 85, 91, 103]. Moreover, net price confidentiality was associated with negative impact on affordability due to risk of substantial overpaying and information asymmetry potentially limiting payers’ purchasing power [93, 112]. Further, it was argued that affordability, financial sustainability, and equitable patient access may be undermined by pricing-related inequities stemming from lower-income EEA member states having to pay relatively high prices despite low absolute prices, and high-income countries (HICs) paying relatively lower – though higher absolute – prices for MPs; however, this may be confounded by parallel trade [33, 52, 76, 78, 81, 82, 103, 153]. Additionally, launch strategies by manufacturers in response to EPR were suggested to lead to launch delays/reluctance and market withdrawals in smaller markets and/or countries with a lower willingness to pay (particularly in Southern and Eastern European countries) [12, 78, 82, 83, 85, 91, 103, 154]. The resulting availability issues, which are further exacerbated by parallel trade incited by low prices in such markets (e.g., Bulgaria) are suggested to substantially impact patient access to MPs [52, 75, 76, 78, 82, 103, 153, 154]. Conversely, positive availability impact was suggested related to countries with little to no price controls at launch (e.g., Germany, UK, Ireland) being favoured for early market launches of MPs [52, 86]. Additionally, literature suggested price convergence towards an international average, or a “race to the bottom”, potentially discouraging innovation due to decreased revenue limiting R&D investments [76, 79, 82, 103, 113]. Similarly, R&D investments regarding future pIHTs may be disincentivised by spill-over effects of EPR and parallel trade [80, 82, 103]. Moreover, EPR was suggested to limit the viability of other price regulation methods – especially VIP – and to lead to path dependence, due to which price levels are influenced by EPR rules across countries rather than other relevant market aspects [78]. Despite these shortcomings, literature suggested wide acceptance and common application of EPR as a cost-containment tool [52, 76]. Its provision of a “benchmark” for domestic price setting and a popular starting point for further price negotiations was highlighted as a major benefit considered by policymakers [79, 112]. However, stakeholders were suggested to consider price inequities across countries resulting from EPR as unacceptable [153]. Furthermore, EPR – especially when involving regular price revisions – was seen as a resource-intensive and technically and administratively complex tool [52, 79, 80, 83]. A more detailed overview is provided in Table 5 and Online Resource 4.

With regard to *CBP*, the included literature suggested that this policy intervention may help disincentivise monopoly price charging and thus increase MP affordability [101, 121]. Feasibility of CBP was suggested to be facilitated through the incorporation of market principles; however, price calculations may involve arbitrariness from manufacturers regarding the inclusion of indirect costs, which would potentially increase prices [122].

According to the included literature, *VIP* involving added therapeutic benefit assessments has been beneficial for ensuring affordability of pIHTs despite their high prices and led to savings and better health budget allocations [57, 58, 73, 96, 97, 103]. France’s practice of revision-based price decreases is suggested to facilitate the financing of expensive MPs [44]. Further, VIP-based price negotiation systems in Germany and France have seen broad acceptance across stakeholders – especially manufacturers – who consider them satisfactory and as a basis for accepting lower prices, respectively [45, 60, 96]. However, especially in strongly differing pricing and reimbursement systems, implementation of a VIP policy may require revisions of coverage and negotiation rules [96]. Moreover, literature suggests that VIP involving pharmaco-economic evaluations may lead to potential affordability issues, requiring payment schemes, though the incentive of a launch deadline following a positive evaluation outcome may motivate threshold-compliant pricing by manufacturers and faster availability (UK) [42, 43]. This pricing policy intervention may further incentivise the development of added-value-generating MPs, though a too low price might be a discouraging factor as innovation would not be sufficiently rewarded [12, 128]. Additionally, the application of cost-effectiveness thresholds in pharmaco-economic evaluations in the UK was suggested to lead to price levels corresponding to the applicable willingness to pay. This was argued to potentially lead to uneven distributions of an MP’s benefit surplus between payers and manufacturers and volatility of R&D returns. [72]. Overall, while the use of pharmaco-economic evaluations underpinning VIP was considered a logical and fair approach to promote access and reward useful innovation, it was viewed as difficult to implement as well as resource- and time-intensive, potentially impairing timely patient access [12]. This particularly applies to therapeutic areas with no alternative treatment and severe life-threatening or debilitating diseases [12].

Based on the information on access-related impact and organisational (dis-)advantages regarding *other policy interventions* [101, 102, 122, 123, 130, 131], we found that the US 340B Pricing Programme was suggested to increase access to high-cost oncology services, especially in rural communities [130]. Furthermore, opinion on whether the Japanese Price Maintenance Premium promoted innovation was divided [101, 123]. However, it was viewed as a reasonable approach for assessing the value of new MPs in clinical practice [102].

#### Pricing implementation methods

Table 6 and Online Resource 5 provide a detailed overview on observed and potential access-related impacts and organisational (dis-)advantages of applied pricing implementation methods according to the included literature. No relevant information was found for OMPs and medical devices.

Based on the included relevant literature [44, 45, 56, 57, 66, 73, 97, 116, 121, 132–135], *price negotiations* are generally suggested to set limits to what is paid for a MP and to potentially improve affordability without delaying availability after market entry (e.g., in Germany) [44, 57, 134]. Failed negotiations and subsequent withdrawals from the German market were not associated with MPs with added therapeutic benefit for at least one indication [45, 56, 57, 73]. However, regarding Medicare negotiations, Hwang et al*.* (2022) saw the potential launch of alternative versions of existing products in order to avoid price negotiations and inflation-linked penalties as a point of concern [135].

With regard to the pricing implementation concerning *MPs for multiple indications*, empirical evidence suggested price decreases of an MP with each follow-on indication (France, Germany). This may account for the lower added clinical benefit of each new indication and the increasing patient population [137]. While multi-indication pricing may potentially decrease payers’ expenditures, measures to protect set prices of high-value indications could diminish that effect [41]. Further, manufacturers may be reluctant to launch MPs for low-value indications, creating availability issues [137]. However, multi-indication pricing was suggested to potentially optimise R&D incentives (especially for high-value secondary indications) and allow manufacturers to capture economic surpluses for each indication [32, 41, 136]. Regarding organisational (dis-)advantages, this implementation method was suggested to be generally viewed critically, potentially facing opposition across stakeholder groups due to implementation complexity and error potential (as observed in Spain and suggested for the UK) [32, 41, 111]. Moreover, separate price negotiations for each indication may lead to a considerable transactional burden for payers [32]. Implementation of multi-indication pricing was further linked to a substantial increase in administrative costs in view of the development and maintenance of suitable data systems [136–138, 141]. Additionally, regulatory constraints and privacy concerns were suggested to constitute feasibility barriers [137, 138].

The relevant literature on access-related impact and feasibility-related (dis-)advantages of *price transparency* [55, 145, 147, 148, 150] suggested that disclosure of negotiated prices was associated with lower prices over time (Switzerland) and more realistic price referencing based on an MP’s added clinical benefit (Germany) [55, 145]. Furthermore, transparency in combination with financial incentives is suggested to reduce MP spending. However, evidence was considered inconclusive in view of disclosure of rebates and discounts following originally confidential payer–manufacturer agreements and improper price reporting [55, 147].

## Discussion

### Main findings

This scoping review pursued two main objectives. First, we aimed to map the existing research regarding determinants of pIHT prices based on stakeholder views as well as pricing policies in EEA/OECD member states, while also depicting the policies’ access-related impact and organisational (dis-)advantages. The second goal involved the detection of knowledge gaps regarding these areas. While we found diverging views across stakeholder groups on relevant price determinants, the included literature focused on value-based determinants. Moreover, there is heterogeneity regarding the applied pricing policies across EEA/OECD member states. Most countries involve a combination of policy interventions and implementation methods. This makes it difficult to isolate their individual effects on patient access to pIHTs.

Our findings indicate that while reference-based pricing, especially EPR, is widely used as a primary policy intervention, it is linked to severe access-related shortcomings. Such negative effects include launch sequencing by manufacturers and price inequities. Since reconciliation with other pricing methods is suggested to be difficult [79], the adoption of different or additional pricing policy interventions to mitigate these effects would therefore require the abolition of EPR or its adjustment to a supportive pricing method (e.g., to inform a maximum price). Conversely, the application of VIP involving an assessment of an MP’s added clinical and/or economic value may be more advantageous for improving patient access. Overall, more complex pricing policies including VIP are viewed more positively by literature and stakeholders [45, 57, 58, 60, 73, 96, 97, 103]. However, adopting and maintaining such policies may require substantial administrative and financial resources, and a considerably long transition period. This may excessively strain many countries’ healthcare budgets and thus reduce positive access-related effects. Additional drawbacks of a VIP-based pricing policy concern the time- and resource-intensive nature of value assessments, and uncertainty regarding the extent of R&D returns [12, 72], which potentially impair acceptability and feasibility of its implementation.

VIP policies employ either assessments on added therapeutic value (e.g., Germany, France) or pharmaco-economic evaluations (e.g., Sweden, UK) to inform price setting and/or price negotiations. In this context, the umbrella term “value-based pricing” might not sufficiently capture the differences of value based on clinical effectiveness compared to value that incorporates economic considerations. This distinction is also adequate in view of the EU HTA Regulation: Joint Clinical Assessments for pIHTs under its scope are strictly limited to comparisons of clinical effectiveness, while the implementation of pharmaco-economic evaluations remains under national jurisdiction [160].

Additionally, differences in pricing policies across countries may influence the affordability and availability of pIHTs and thus, patient access. Based on our findings, the literature suggests that pricing policies rooted in EPR, which are particularly applied in Southern and Eastern European countries, might disadvantage these markets. This is often associated with manufacturers’ launch strategies as well as relatively higher prices in view of the economic capacities of such countries’ healthcare systems. While this might suggest VIP as a more favourable policy intervention regarding patient access, differences in time to access are also driven by further factors that are subject to cross-country heterogeneity (e.g., attractiveness of markets allowing high prices, duration of HTA and reimbursement procedures, time of market authorisation) [161–163].

Especially across EEA member states, pricing policy aspects are often subject to Change. For instance, in 2023, Germany Reduced the free-pricing period following market launch from 12 to six months [47, 73]. Further, in countries applying EPR, reference countries can either be added to, removed from, or changed in the respective baskets. For instance, the Netherlands has replaced Germany with Norway as a reference country [164]; and Switzerland increased the reference basket size [103]. Further adjustments of a reference basket size are based on changes of the member states of the Eurozone (e.g., Croatia joining in 2023), or of the EU/EEA itself (e.g., the UK leaving the EU in 2019). This may also explain the observed reporting inconsistencies among EPR-focused records in this regard.

### Knowledge gaps

Our results indicate that literature on stakeholder views regarding price determinants was scarce. Most literature on price determinants appears to either be underpinned by economic theory, or by purely academic or statistical considerations [72, 165–167]. Only one record presented information based on stakeholder view elicitation [28]. Hence, there is scope for eliciting stakeholder preferences and views on pIHT price determinants and their prioritization, which can provide valuable input for the development of access-oriented pricing models and pricing policy design. Additionally, such further research might help confirm or refute the findings from the relevant included literature.

Moreover, the amount of available information regarding applied pricing policies differed per country. While more detailed information was available for high-income countries, EEA/OECD member states with a lower income level (Costa Rica, Chile) or (very) small markets (e.g., Iceland, Liechtenstein, Malta) were, at most, sparsely featured. Further research may help provide a more detailed picture regarding pricing policies and their access-related impact in such countries.

We found scant evidence on price determinants and pricing policies for medical devices. A possible explanation for this is that prices of medical devices are often arranged between manufacturer and healthcare provider, or freely set by the manufacturer [168–170]. Generally, there appears to be no consistent application of medical device pricing at a governmental or legislative level. Therefore, when developing a pricing model tailored to specific medical device types, the interests of its users should be carefully considered. Finally, information related to environmental sustainability was scarce. For the pricing of cell therapies, France *inter alia* considers the manufacturer’s domestic footprint [64]. Based on the collected information, this is not done in any other EEA/OECD member state nor for any other pIHT. Further research could investigate to what extent this price determinant has influenced the price of reimbursed cell therapies in France. Such information may also be useful for the development and implementation of pricing models that also consider environmental sustainability in connection with pIHTs. This is also relevant in view of environmental sustainability playing a vital role in the proposed EU pharmaceutical legislation reform [171].

### Strengths and limitations

This review makes several contributions to the literature. Firstly, it focuses on stakeholder views regarding pIHT-related price determinants. This contrasts a review by Borges Dos Santos et al*.* involving a theoretical and quasi-experimental approach regarding pricing models [172], as well as research by Vogler et al. eliciting stakeholder views on pricing policies [173]. Secondly, this review provides a comprehensive overview on access-related impacts and organisational (dis-)advantages of applied pricing policies across EEA/OECD member states. While previous reviews in this regard were restricted to individual policy interventions, such as EPR [52, 78, 103], this study consolidates relevant findings across multiple policy interventions and price implementation methods.

However, like other studies, this scoping review has limitations. First, we decided not to include literature on theoretical considerations regarding pIHT price determinants and their use in price calculations. This exclusion also concerned pricing models developed in an academic/theoretical setting and discussed in recent literature [174], such as Uyl-de Groot & Löwenberg’s cost-based cancer-treatment pricing model [175] or Nuijten et al.’s discounted cash-flow model [176], and may have provided a narrow picture of the integration of price determinants for setting a pIHT price. Taking economic theory and practical application in pricing policies into consideration may have provided a more complete picture of relevant pIHT prices determinant. Second, only publications in English were considered. This might have been a restricting factor to detecting relevant literature, especially policy papers. Including records in more languages, such as Spanish, might have provided us with more relevant publications and thus more detailed information on Latin American countries. However, we believe that these findings would not substantially change the main findings of this review. Third, pricing policy interventions and implementation methods applied in EEA/OECD member states were considered at the time of publication of the included records. Given the previously mentioned changes of pricing policy aspects, this may have led to potential inconsistencies in our findings.

Fourth, access-related impacts of pricing policies may be confounded by other factors, such as reimbursement policies, market authorisation regulations (especially outside the EEA), market mechanisms like parallel trade, and ageing patient populations [102]. This may over- or underestimate the isolated effect of pIHT pricing on affordability, availability, and equity for payers and patients, respectively. Further research is required to adjust for potentially confounding variables to discern the isolated effect of pricing policies on patient access in individual countries. Fifth, in countries employing VIP-based policies, it is difficult to fully delineate pricing-related practices from reimbursement-related aspects with regard to healthcare decision-making procedures since these are often interwoven with each other. While this study’s search strategy and reviewing process focused on including information relevant to pricing policies exclusively, the completeness of our findings might be impaired by potentially missing reimbursement-related aspects that may influence MP pricing. Nonetheless, with the other described limitations as a caveat, we believe that our findings accurately represent the applied pricing policies across EEA/OECD member states, given the consistency of information on applied pricing policy interventions and implementation methods encountered in the included literature.

Finally, the focus of this review concerned pricing policies applied in individual countries; pricing based on international collaborations was not considered. While we acknowledge the importance of such collaborations between numerous countries within the EEA (e.g., Beneluxa, Valletta Declaration, Baltic Procurement Initiative), successful joint negotiations have so far been rare (e.g., reimbursement of Spinraza® [nusinersen] in Belgium and the Netherlands [177]). Therefore, regarding successful outcomes, the practical relevance of such collaborations for pricing has so far been negligible and their inclusion would not have led to different findings in our study. Nonetheless, despite inherent challenges to such partnerships, successful collaborations may encourage governments to establish and maintain such partnerships, and manufacturers to engage in joint negotiations.

## Conclusion

This scoping review shows that pIHT-related pricing policies in EEA/OECD member states often involve combinations of policy interventions and implementation methods, with EPR and price negotiations being prominently applied. The application of such combinations makes it difficult to estimate the individual policy interventions’ effect on patient access. While the literature suggests severe access-related shortcomings of EPR, VIP is generally viewed more favourably. However, the latter is accompanied by inherent disadvantages regarding feasibility and resource use. Further research should aim to elicit stakeholder viewpoints regarding relevant price determinants and their prioritisation and integration for pIHT price calculations, and the involvement of environmental aspects in price setting. This may provide valuable input for the development of pricing models aiming to improve patient access to pIHTs. Furthermore, based on our findings, a transition from reference-based pricing to policies employing multiple interventions including VIP might help decision-makers to balance innovation support, equitable patient access, and financial sustainability of healthcare systems.

## Supplementary Information

Below is the link to the electronic supplementary material.

Supplementary file1 (DOCX 33 KB)

Supplementary file2 (DOCX 20 KB)

Supplementary file3 (DOCX 20 KB)

Supplementary file4 (DOCX 90 KB)

Supplementary file5 (DOCX 50 KB)

## Data Availability

All data generated or analysed within this study are included in this article, the corresponding references, and the supplementary appendix.
