# Supplementary material for: Price determinants and pricing policies concerning potentially innovative health technologies: a scoping review
Source: Eur J Health Econ. 2025 Sep 6;27(2):479–508. doi: 10.1007/s10198-025-01834-y (PMC13046678; doi:10.1007/s10198-025-01834-y)
Supplement: Supplementary file 1 — Supplementary file1 (DOCX 33 KB) [file 10198_2025_1834_MOESM1_ESM.docx]

# Online Resource 1: Search Strategies

## Scientific literature databases

Table S1-1: Hits provided by search strategies

| **Database searched** | **Platform** | **Years of coverage** | **Records** | **Records after duplicates removed** |
| --- | --- | --- | --- | --- |
| Medline ALL | Ovid | 1946 - Present | 4096 | 4072 |
| Embase | Embase.com | 1971 - Present | 5255 | 1823 |
| Web of Science Core Collection* | Web of Knowledge | 1975 - Present | 2475 | 391 |
| Additional Search Engines: Google Scholar** | | | 150 | 122 |
| Total | | | 11976 | 6408 |

*Science Citation Index Expanded (1975-present) ; Social Sciences Citation Index (1975-present) ; Arts & Humanities Citation Index (1975-present) ; Conference Proceedings Citation Index- Science (1990-present) ; Conference Proceedings Citation Index- Social Science & Humanities (1990-present) ; Emerging Sources Citation Index (2005-present)

*Exact search turned on in Web of Science Core Collection

**Google Scholar was searched via "Publish or Perish" to download the results in EndNote.

No other database limits were used than those specified in the search strategies

Articles excluded before 2014: 2546

**Medline**

(Technology, High-Cost / OR ((Drug Development/) AND (Drug Costs/)) OR (((high-cost* OR high-price* OR ultra-cost* OR ultra-price* OR expensive* OR costly OR price* OR pricing*) ADJ6 (technolog* OR medicine* OR treatment* OR therap* OR intervent* OR drug* OR agent* OR device* OR innovat* OR pharmaceutical*)) OR ((high*-tech* OR high*-innovat*) ADJ6 (medicine* OR treatment* OR therap* OR intervent* OR pharmaceutical*)) OR ((cost OR price) ADJ6 (drug* OR medicine* OR pharmaceutical*) ADJ6 (develop* OR research OR new OR determinant*))).ab,ti,kw. OR (((cost OR price*) AND (drug* OR medicine* OR pharmaceutical*) AND (develop* OR research OR new OR determinant*))).ti.) AND (* Cost Control / OR * Health Policy / OR (pricing).ab,ti,kw. OR (cost-control* OR affordab* OR sustainab* OR budget* OR access-to OR barrier* OR ((cost OR costs OR price* OR resource-us*) ADJ6 (calculat* OR how-much OR integrat* OR compar* OR factor* OR policy* OR policies* OR decision*))).ti.)

**Embase**

('high-cost technology'/de OR (('new drug'/de OR 'drug development'/de) AND ('drug cost'/de)) OR (((high-cost* OR high-price* OR ultra-cost* OR ultra-price* OR expensive* OR costly OR price* OR pricing*) NEAR/6 (technolog* OR medicine* OR treatment* OR therap* OR intervent* OR drug* OR agent* OR device* OR innovat* OR pharmaceutical*)) OR ((high*-tech* OR high*-innovat*) NEAR/6 (medicine* OR treatment* OR therap* OR intervent* OR pharmaceutical*)) OR ((cost OR price) NEAR/6 (drug* OR medicine* OR pharmaceutical*) NEAR/6 (develop* OR research OR new OR determinant*))):Ab,ti,kw OR (((cost OR price*) AND (drug* OR medicine* OR pharmaceutical*) AND (develop* OR research OR new OR determinant*))):ti) AND (affordability/de OR 'cost control'/mj OR 'health care policy'/mj OR (pricing):ab,ti,kw OR (cost-control* OR affordab* OR sustainab* OR budget* OR access-to OR barrier* OR ((cost OR costs OR price* OR resource-us*) NEAR/6 (calculat* OR how-much OR integrat* OR compar* OR factor* OR policy* OR policies* OR decision*))):ti) NOT ([conference abstract]/lim AND [2000-2020]/py)

**Web of science**

(TS=(((high-cost* OR high-price* OR ultra-cost* OR ultra-price* OR expensive* OR costly OR price* OR pricing*) NEAR/5 (technolog* OR medicine* OR treatment* OR therap* OR intervent* OR drug* OR agent* OR device* OR innovat* OR pharmaceutical*)) OR ((high*-tech* OR high*-innovat*) NEAR/5 (medicine* OR treatment* OR therap* OR intervent* OR pharmaceutical*)) OR ((cost OR price) NEAR/5 (drug* OR medicine* OR pharmaceutical*) NEAR/5 (develop* OR research OR new OR determinant*))) OR TI=(((cost OR price*) AND (drug* OR medicine* OR pharmaceutical*) AND (develop* OR research OR new OR determinant*)))) AND (TS=(pricing) OR TI=(cost-control* OR affordab* OR sustainab* OR budget* OR access-to OR barrier* OR ((cost OR costs OR price* OR resource-us*) NEAR/5 (calculat* OR how-much OR integrat* OR compar* OR factor* OR policy* OR policies* OR decision*)))) AND TS=(healthcare OR health-care OR hospital* OR medicin* OR drug*) AND DT=(article)

**Google scholar**

"high|highly|ultra cost|price|technology|innovative technology|medicine|medicines|treatment|therapy|intervention|drugs|agents|devices|innovation"|"costly|expensive medicine|treatment|therapy|intervention" pricing|intitle:"cost|budget control|affordable|affordability"

## Grey literature searches

**BASE: 324 hits with restricted source types: Book (incl. book part), conference object, report, review, manuscript, doctoral/postdoctoral thesis**

(price pricing "high cost" "high price" expensive) AND (technology medicine treatment intervent* drug agent therapy device innovat*) AND (affordability access* budget "cost control" "health care policy" "healthcare policy" policy decision*) AND (EU EEA OECD Iceland Norway Sweden Finland Estonia Latvia Lithuania Poland Germany Netherlands Belgium Luxembourg "United Kingdom" Britain Ireland France Switzerland Liechtenstein Austria Spain Portugal Italy Malta Slovenia Croatia Czechia "Czech Republic" Slovakia Hungary Romania Bulgaria Greece Cyprus Canada Turkey "United States" America Japan Australia "New Zealand" Mexico "South Korea" Chile Israel Colombia "Costa Rica") subj:(health "health policy") doctype:(11* 13 14 15 183 19) lang:en year:[2014 TO 2023]

**CORDIS: 51 hits**

('health technolog*' AND (new OR innovati*)) AND (price* OR pricing) AND (policy* OR policies) AND (affordability OR access OR decision*) AND (/article/relations/categories/collection/code='resultsPack','projectsInfoPack','brief','news' OR (/result/relations/categories/collection/code='deliverable','publication','exploitable' OR (/result/relations/categories/collection/code='pubsum' OR contenttype='programme'))) AND applicationDomain/code='health' AND language='en' AND contentUpdateDate>=2014-01-01

**ISPOR – search focus on specific sources (News, Value Outcomes Spotlight): 165 hits**

(pricing OR price* OR "high price" OR "high cost" OR expensive OR costly) AND ("health technolog*" OR medicine OR treatment OR intervent* OR drug* OR agent* OR therap* OR device* OR innovat*) AND (affordability OR access*) AND (policy OR policies) NOT "value in health"

***Remark:*** *Results from Value in Health are already part of the scientific literature search and therefore did not require a separate search on ISPOR. Websites (831 results) were considered to be too unspecific and to create too much noise.*

**NICE:** **68 hits**

(pricing OR price) AND ("health technology" OR medicine OR drug OR pharmaceutical OR "medical device") AND (policy OR policies)

**ICER: 27 hits**

No specific search terms applied; only policy papers were considered. Assessments (which were all disease-specific; 104 hits) were disregarded since they are outside of the scope of the review questions concerning pricing policies).

**OECD: 137 results**

(Abstract ‘price’) (Language ‘en’) **OR** (Abstract ‘pricing’) **AND** (Abstract ‘"health technolog*"’) **OR** (Abstract ‘drug*’) **OR** (Abstract ‘pharmaceutic*’) **AND** ( ‘’) **AND** (Theme ‘Social Issues/Migration/Health’) **OR** (Abstract ‘"medical device*"’) **AND** (Abstract ‘new’) **OR** (Abstract ‘innovat*’) **From Theme** Social Issues/Migration/Health  published between 2014 and 2023

**EFPIA: 13 hits**

https://efpia.eu/search-results/?query=pricing; https://efpia.eu/search-results/?query=price

***Remark:*** *15 results were found for “pricing”, 4 for “price”. 6 of those results were out of the time-related scope and therefore not included in the screening process.*

**AIM: 46 hits**

https://www.aim-mutual.org/?s=pricing https://www.aim-mutual.org/?s=price https://www.aim-mutual.org/?s=access

**BEUC (European Consumer Association): 52 hits** https://www.beuc.eu/search?parameter=pric*%20%22health%20technolog*%22&created[min]=2014-01-01&created[max]=2023-08-31&field_priority[2943]=2943

**PhRMA: 153 hits**

https://phrma.org/resource-center/Search#refresh-search_e=0&refresh-search_refreshcontenttypes=Policy%20Paper%2CPress%20release%2CReport%2CResearch%20In%20Your%20Backyard%2CFact%20Sheet%2CCodes%20%C3%96%20Guidelines%2CBlog&refresh-search_refreshtopics=Price%20Setting%2CAccess%20to%20Medicines

**WHO (restricted to Publications): 22 hits** https://www.who.int/home/search?indexCatalogue=genericsearchindex1&searchQuery=pricing&wordsMode=AllWords

https://www.who.int/home/search?indexCatalogue=genericsearchindex1&searchQuery=price&wordsMode=AllWords

**Health Action International: 41 hits**

https://haiweb.org/publicationsarchive/?_sf_s=pricing

**Google Advanced Search: first 100 hits – 94 hits importable**

Same search as with Google Scholar (cf. above).

## Deduplication / Merging with Zotero

Table S1-2: Results of deduplication and merging process

| **Database** | **Original no. of results** | **No. of duplicates removed** | **No. of results after deduplication** |
| --- | --- | --- | --- |
| Scientific literature databases | 3,862 | 4 | 3,858 |
| AIM | 46 | 17 | 29 |
| BASE | 324 | 169 | 155 |
| BEUC | 52 | 1 | 51 |
| CORDIS | 51 | 1 | 50 |
| EFPIA | 13 | 0 | 13 |
| Google Adv. Search | 94 | 11 | 83 |
| Health Action Int’l | 41 | 0 | 41 |
| ICER | 27 | 1 | 26 |
| ISPOR | 165 | 0 | 165 |
| NICE | 68 | 2 | 66 |
| OECD | 137 | 0 | 137 |
| PhRMA | 152 | 2 | 150 |
| WHO | 22 | 0 | 22 |
| *Total results scientific literature* | *3,862* | *4* | *3,858* |
| *Total results grey literature* | *1,192* | *204* | *988* |
| ***Total results*** | ***5,054*** | ***208*** | ***4,846*** |

Abbreviations: Adv., advanced; AIM, Association Internationale de la Mutualité/International Association of Mutual Benefit Societies; BASE, Bielefeld Academic Search Engine; BEUC, Bureau Européen des Unions de Consummateurs/European Consumer Organisation; CORDIS, Community Research and Development Information Service; EFPIA, European Federation of Pharmaceutical Industries and Associations; ICER, Institute for Clinical and Economic Review; Int’l, international; ISPOR, International Society for Pharmacoeconomics and Outcomes Research; NICE, National Institute for Health and Care Excellence; no., number; OECD, Organisation for Economic Co-operation and Development; PhRMA, Pharmaceutical Research and Manufacturers of America; WHO, World Health Organization.

## Manual filtering by publication year: exclusion of publications before 2014

Table S1-3: Results of filtering process

| **Database** | **Original no. of results after deduplication** | **No. of results published before 2014 removed** | **No. of results after filtering** |
| --- | --- | --- | --- |
| Scientific literature databases | 3,858 |  |  |
| AIM | 29 | 0 | 29 |
| BASE | 155 | 61 | 94 |
| BEUC | 51 | 0 | 51 |
| CORDIS | 50 | 6 | 44 |
| EFPIA | 13 | 0 | 13 |
| Google Adv. Search | 83 | 1 | 82 |
| Health Action Int’l | 41 | 1 | 40 |
| ICER | 26 | 0 | 26 |
| ISPOR | 165 | 0 | 165 |
| NICE | 66 | 0 | 66 |
| OECD | 137 | 0 | 137 |
| PhRMA | 150 | 0 | 150 |
| WHO | 22 | 0 | 22 |
| *Total results scientific literature* | *3,858* | *0* | *3,858* |
| *Total results grey literature* | *988* | *69* | *919* |
| ***Total results*** | ***4,846*** | ***69*** | ***4,777*** |

Abbreviations: Adv., advanced; AIM, Association Internationale de la Mutualité/International Association of Mutual Benefit Societies; BASE, Bielefeld Academic Search Engine; BEUC, Bureau Européen des Unions de Consummateurs/European Consumer Organisation; CORDIS, Community Research and Development Information Service; EFPIA, European Federation of Pharmaceutical Industries and Associations; ICER, Institute for Clinical and Economic Review; Int’l, international; ISPOR, International Society for Pharmacoeconomics and Outcomes Research; NICE, National Institute for Health and Care Excellence; no., number; OECD, Organisation for Economic Co-operation and Development; PhRMA, Pharmaceutical Research and Manufacturers of America; WHO, World Health Organization.
