# Supplementary material for: Price determinants and pricing policies concerning potentially innovative health technologies: a scoping review
Source: Eur J Health Econ. 2025 Sep 6;27(2):479–508. doi: 10.1007/s10198-025-01834-y (PMC13046678; doi:10.1007/s10198-025-01834-y)
Supplement: Supplementary file 2 — Supplementary file2 (DOCX 20 KB) [file 10198_2025_1834_MOESM2_ESM.docx]

# Online Resource 2: Exclusion criteria for Title-Abstract Screening and Full-Text Review

Table S2-1: Exclusion criteria applied to the title-abstract screening

| 0 – publication <2014 |
| --- |
| 1 – not in English |
| 2 – not about existing pricing methods/policies |
| 3 – not about pIHTs in general |
| 4 – not about on-patent/repurposed pIHTs |
| 5 – not concerning EEA/OECD countries |
| 6 – to be included |

Abbreviations: EEA, European Economic Area; OECD, Organisation for Economic Co-operation and Development; pIHT, potentially innovative health technology.

Table S2-2: Exclusion criteria applied to the full-text review

| 00 – record not retrievable |
| --- |
| 01 – publication <2014 |
| 02 – publication not in English |
| 03 – publication consists of abstract only |
| 04 – publication is a conference paper |
| 05 – publication is comment/reply to previous publication |
| 06 – publication is an opinion paper |
| 07 – not concerning EEA/OECD countries |
| 08 – not about existing pricing methods/policies as main focus |
| 09 – no stakeholder considerations re pricing methods involved |
| 10 – not about pIHTs in general |
| 11 – not about on-patent/repurposed pIHTs (publication concerns generics/biosimilars) |
| 12 – pricing policy in record is outdated |
| 13 – to be included |

Abbreviations: EEA, European Economic Area; OECD, Organisation for Economic Co-operation and Development; pIHT, potentially innovative health technology.
