# Supplementary material for: Price determinants and pricing policies concerning potentially innovative health technologies: a scoping review
Source: Eur J Health Econ. 2025 Sep 6;27(2):479–508. doi: 10.1007/s10198-025-01834-y (PMC13046678; doi:10.1007/s10198-025-01834-y)
Supplement: Supplementary file 3 — Supplementary file3 (DOCX 20 KB) [file 10198_2025_1834_MOESM3_ESM.docx]

# Online Resource 3: Data Extraction Instrument

1. (First) author
2. Year of publication
3. Title of publication
4. Type of literature
5. Funding / author affiliation (stakeholder groups, perspectives of the (leading) authors)
6. Key objectives
7. Country of origin of study (where the study in the source was conducted)
8. Type of health technology
   1. Select type of health technology (pharmaceutical/medical device)
   2. Specify type of health technology
9. Therapeutic area
10. Country of focus/policy
11. Price determinants
    1. Cost-based determinants
       1. Cost-based? (yes/partially/no)
       2. Description of cost-based determinants
    2. Value-based determinants
       1. Value-based? (yes/partially/no)
       2. Description of value-based determinants
    3. Price referencing
       1. Reference-based? (yes/partially/no)
       2. Description of referencing
    4. Other price determinants
       1. Other determinants? (yes/partially/no)
       2. Description of other determinants
12. Applied pricing policies on potentially innovative health technologies
13. Transparency as an element of pricing policies (yes + extent/no)?
14. Impact of pricing policies in practice
    1. Impact on affordability
    2. Impact on availability
    3. Impact on sustainability
    4. Impact on equity
    5. Other impact
15. Organisational advantages/disadvantages of applied pricing policies
    1. Acceptability
    2. Resource use
    3. Feasibility
