# Supplementary material for: Price determinants and pricing policies concerning potentially innovative health technologies: a scoping review
Source: Eur J Health Econ. 2025 Sep 6;27(2):479–508. doi: 10.1007/s10198-025-01834-y (PMC13046678; doi:10.1007/s10198-025-01834-y)
Supplement: Supplementary file 4 — Supplementary file4 (DOCX 90 KB) [file 10198_2025_1834_MOESM4_ESM.docx]

# Online Resource 4: Access-related impact and organisational (dis-)advantages of applied quantitative pricing policy interventions – detailed overview

Table S4-1: Sources of evidence on access-related impact and organisational (dis-)advantages of applied quantitative pricing policy interventions in EEA/OECD member states (detailed overview)

| **Policy intervention** | **References** | **Description of impact^1^** | **Description of (dis-)advantages^2^** |
| --- | --- | --- | --- |
| Free pricing | [35, 43–45, 48, 49, 65, 71] | **Affordability:**  DK:   - Strong competition through frequent price changes, thus lower MP prices   US:   - Payment-related impact (increased deductibles and co-payments); for specialty MPs, patients must pay coinsurance. - Circumvention of co-payment system by manufacturers through coupon system to reimburse OOP expenses and facilitate affordability for patients. - Free pricing has enabled originator price growth exceeding inflation (rising launch prices, price increases). - Substantially higher prices in comparison to neighbouring countries. - High prices have detrimental effect on affordability for payers and patients.   **Availability:**  *DE:*   - Free pricing at launch ensures rapid access.   *FR:*   - TAU allows market launch of highly innovative MPs before price negotiation at price chosen by manufacturer.   *US:*   - Higher prices make MPs more likely to be available first in the US. - States with higher MP costs for Medicaid had to cut services or increase eligibility requirements   **Sustainability:** NA  **Equity:**  *US:*   - Uninsured patients cannot afford expensive MPs. - High prices of orphan MPs limit patient access.   **Other impact:**  *US:*   - Increased deductibles and co-payments/coinsurance may reduce use of/adherence to effective medications. - Divergent growth of US MP prices implies increasing strain on reimbursement negotiations outside the US if manufacturers seek to maintain income-related differentials. - Novel products may be utilised more. | NA |
| Reference-based pricing | [10, 12, 33, 52, 75, 76, 78–86, 91, 93, 96, 101, 103, 105, 108, 112, 113, 115, 120, 153–155] | **Affordability:**  *General:*   - EPR: No substantial reduction in international price differences has occurred within EU countries; some reduction suggested on global scale. - EPR: can lead to substantial savings for public payers (short-term). - EPR: decrease of net prices for orphan MPs suggested. - EPR reducing manufacturers’ willingness to price to market leads to affordability problems. - EPR: Pharmaceutical companies may increase target price to avoid negative impact on revenues and parallel trade. - EPR: Referencing official list prices instead of adjusting for discounts/rebates leads to risk for payers of (substantial) overpaying. - EPR: information asymmetry limits payers’ purchasing power (also for potential follow-up negotiations). - EPR: companies might respond by reducing price transparency (offering discounts/rebates to reference country payers) - EPR: Lack in pricing-related equity undermines affordability – low relative price levels in countries with high absolute prices levels and vice versa – might also partially be explained by parallel trade; also involves innovative and highly efficacious MPs and OMPs. - EPR: price instability as cross-border spill-over effect. - EPR: when used to define price ceilings, it may inhibit further price reduction below the ceiling. - EPR: if implementation does not align with public policy objectives, may influence price levels and lead to inflated prices in LICs, jeopardising affordability. - EPR: irregular monitoring might lead to price reductions in reference countries not automatically being translated into price decreases in referencing country. - IPR may reduce MP prices.   *BG:*   - EPR made MP market low-price. - EPR: Led to decrease of price of reimbursed MPs.   *CA:*   - EPR/IPR: change of reference basket, comparison of costs of more expensive MPs with patient outcomes may lead to lower prices for new branded MPs.   *CO:*   - EPR: Fast decrease of prices.   *EE:*   - EPR: no detectable affordability issues.   *FR:*   - EPR as policy component disincentivises line extension/new indication approval during first 5 years.   *GR:*   - EPR: Lower MP prices after implementation (9.5% in 2010).   *HR:*   - EPR: Launch strategies by manufacturers may increase prices in countries that (in-)directly reference them. - Price reductions in one reference country may not automatically apply to other reference countries, reducing potential savings (unless there are rapid assessment mechanisms).   *HU:*   - EPR: Price decreases in country basket could not be reflected in reimbursement system since EPR is only used at launch.   *IE:*   - EPR: Relatively high prices due to reference basket and calculation method.   *LV:*   - EPR: no detectable affordability issues.   *NL:*   - EPR: considerably lower prices in general.   *RO:*   - EPR: Romanian prices are among cheapest in EU.   *SK:*   - EPR: Expected price reductions of €75 million by 2012.   *TR:*   - EPR: Considerable price reductions   **Availability:**  *General:*   - EPR: Manufacturers may refrain from launching product in a country only accepting low prices (not to jeopardise product’s prices in other market), apply launch sequence strategies to avoid launching new MPs in countries with potential lower prices, lower market volume, strict price setting regulations (contribution of EPR itself unclear, as parallel trade may also contribute to this), or change pricing strategies. Launch delays limit access to innovative and potentially life-saving treatments. - EPR: launch strategies prioritise early entry in DE, UK (countries without direct price controls). - EPR: Price revisions in one country may trigger sequence of circular price revisions, further contributing to strategic launching. - EPR: if WTP is similar across countries: likely not to cause launch delays. - EPR: Sizes of reference country baskets increasing might lead to countries with low WTP not being served and is associated with some price convergence. - EPR: Availability in European LIC negatively impacted; conversely, accelerated access in high-GDP EU countries. - EPR: parallel trade subjects EU MS more to spill-over effects compared to non-EU countries. - EPR: withdrawals from market due to unsatisfactory price levels possible. - IPR: may lead to delayed launch of new products by manufacturers wary to accept a low price.   *BE:*   - EPR: Systematic dossier submission delay by manufacturers to avoid lower prices that would affect other countries in Europe.   *BG:*   - EPR: Withdrawal of products from the market due to low reference prices creates shortages of certain MPs. - EPR: Strategy of referring to the ex-factory manufactory prices leads to serious difficulties in the supply of medicinal products. - EPR: 200 products have been withdrawn since 2012 due to low prices attained by EPR.   *GR:*   - EPR: Launch delays may lead to access issues.   *HR:*   - EPR: No automatic price reductions for new MPs should enhance desire to launch MPs in HR.   *IE:*   - EPR: MPs are on the market relatively early.   *PT:*   - EPR: led to longer launch delays.   *RO:*   - EPR: More than 1175 MPs have disappeared from the market (impact of both pricing and reimbursement mechanism) - EPR: Parallel trade causes some MPs to leave the country.   *SK:*   - EPR: Expansion of reference basket resulted in companies disregarding newly implemented prices or lobbying for exemptions   *TR:*   - EPR; Manufacturers are reluctant to launch new MPs in TR   **Sustainability:**  *General:*   - EPR and IPR: can contribute to cost-containment in the short term; effect appears to decrease over time; focus on short-term financial gains could negatively impact healthcare systems in the long run (as a result of ‘path dependence’). - EPR: regular price revisions can lead to greater short-term cost containment due to lower price levels.   *BG:*   - EPR: Only partial effect on cost-containment due to increasing number of MPs in PDL, increased reimbursement, increased number of patients entitled to reimbursement.   *British Columbia (CA):*   - IPR: savings of CA$161 million in first six years after implementation against total budget of CA$3.07 billion.   *CO:*   - EPR: MP health expenditure almost doubled since introduction.   *GR:*   - EPR: proved ineffective in the long run (expenditure continued to rise at similar levels compared to before introduction).   *SK:*   - EPR: 25% reduction in MP expenditure as proportion to total healthcare spending.   *TR:*   - EPR: Achieved savings of around US$1 billion   **Equity:**  *General:*   - EPR: MP prices remain higher in countries with lower absolute price levels compared to countries with higher absolute price levels; may undermine equitable patient access. Further consequence: such countries may face several years of excessive expenditure. - EPR: no evidence regarding isolated effect on social welfare   **Other impacts:**  *General:*   - EPR: real prices often remain unknown due to confidential discounts (especially for high-priced MPs). - EPR: no access improvement for oncology MPs, due to higher confidential discounts. - EPR: low price in a given market might affect manufacturer’s pricing strategies elsewhere, lead to parallel trade. - EPR reduces manufacturer’s willingness to price to market, contribution to access problems. - EPR with small reference country baskets leads to relatively low losses for seller; lower profits with larger baskets (increased constraints on international prices). - EPR: spill-over effects (due to EPR and parallel trade) likely to lead to access issues and limited benefits to payers and patients in terms of cost-savings for high price markets, and to negative impact on willingness/potential/capacity to invest in R&D. - EPR: (downward) price convergence towards international average; arguably race-to-the-bottom if lowest price in reference basket is used; consequently, potential discouragement of incremental innovation (reduced revenue leading to reduced potential for R&D investment). - EPR: limits ability of other methods to regulate MP pricing; does not take into account role of VIP (also undermines VIP). - EPR: Frequent price revisions may distort role of the market, reduce predictability and produce errors, especially with large reference baskets. - EPR: path dependence, observed price levels are influenced by EPR rules in individual countries, ignoring other market aspects (health needs, income, healthcare costs, extent of variations between countries). - EPR: Exchange rate volatility can affect prices denominated in local currencies. - EPR: Consultant forms support manufacturers in exploiting differences in price-setting algorithms and EPR baskets to explore strategies for launches.   *BG:*   - EPR: Lowest prices of prescription MPs lead to significant problems parallel trade, affecting patients’ access to treatment. - EPR: Unattractive market to sellers of latest new MPs.   *CH:*   - EPR: price cuts reduced industry revenue in CH by €430 million, by €495.2 million worldwide.   *CO:*   - EPR: may spur increase in demand of regulated products, defying cost control objective.   *GR:*   - EPR: failed to control MPs consumption.   *HR:*   - EPR: Using undiscounted average comparable price of 100% for originator MPs enables fair market competition based on such price.   *NZ:*   - IPR: allows for potential formation of cross-product agreements (manufacturer will agree to lower price of another product within a reference group, thus lowering reimbursement price for that group, in exchange for coverage for a new product). | **Acceptability:**  *General:*   - EPR is a widely accepted and commonly applied cost-containment tool. - EPR providing an indication of ‘benchmark’ prices is considered major benefit by several policy-makers. - EPR: popular starting point for further price negotiations. - EPR: inequity between high- and low-GDP countries resulting from EPR regulations considered unacceptable.   *HR:*   - EPR: Price calculation for new MPs appears acceptable to all key stakeholder groups.   **Resource use:**  *General:*   - EPR: technically and administratively more complex process than VIP, requires large amounts of price data. - EPR: Cost- and time-intensive in application.   **Feasibility:**  *General:*   - EPR: difficult to implement, due to lack of available information on MPs’ prices: limited access, price heterogeneity, confidentiality of discounts/rebates, irregular price revisions, exchange rate volatility, price reductions are not automatically translated. - EPR: does not require great investments in HTA and pharmaco-economics as would be the case for VIP. - EPR: Identification of same MP in other countries can be challenging (different commercial names, formulations, dosages, pack sizes). - EPR: using ex-factory price is considered more suitable than wholesale price due to different distribution margins and tax rates across countries. - EPR: described as inefficient approach to reducing prices when used in isolation.   *BG:*   - EPR: Workload for price revisions and their publication too large to keep up with the statutory terms. |
| Cost-based pricing | [101, 121, 122] | **Affordability:**  *General:*   - Helps protect patient populations with rare diseases from manufacturers dominating the market and wishing to charge monopoly prices. - Empirical impact uncertain.   **Availability:**  *General:*   - Empirical effect uncertain.   **Sustainability:** NA  **Equity:** NA  **Other impacts:**  *General:*   - Costs of failed R&D efforts might not be recovered, which could adversely influence investments | **Acceptability:** NA  **Resource use:** NA  **Feasibility:**  *JP:*   - Calculation of manufacturer’s indirect expenses is subject to inevitable arbitrariness on part of manufacturer (overhead costs can increase depending on their intention). |
| Value-informed pricing | [12, 42–45, 57, 58, 60, 72, 73, 96, 97, 103, 111, 128] | **Affordability:**  *DE:*   - AMNOG resulted in relative decrease in incremental treatment costs between launch and price negotiation (24.5%). - Prices are among the highest among European countries, relatively low price levels. - DE approach takes into account high- and low-value indications; perceived to be the best way to ensure affordability of novel therapies.   *FR:*   - Added value approach saves money (compared to US).   *UK*:   - Potential affordability issues despite cost-effectiveness given the allocated healthcare budget.   **Availability:**  *DE:*   - No withdrawals observed for MPs with added benefit for at least one patient group of an indication. - AMNOG did not significantly affect patient’s access; price negotiation after market entry should not delay patient access. - Availability is among the highest in Europe.   *UK:*   - Incentive of 90-day deadline for launch following positive NICE recommendation incentivises threshold-compliant pricing; thus, faster availability; access is automatic.   **Sustainability:**  *DE:*   - Savings of €14 billion for health insurance funds accumulated 2011–2021. - Uncertainty of effect on prescription MP expenditure.   *FR:*   - Added value approach allocates funds better (compared to US). - Implementation of policy has stopped MP spending growth without slowing access to innovative MPs.   *KR:*   - Policy has worked reasonably well for lowering MP prices.   **Equity:**  *FR:*   - Savings from price decreases of older MPs facilitate financing of innovative and expensive new MPs. - Implementation of policy has stopped MP spending growth without slowing access to innovative MPs.   **Other impacts:**  *General:*   - Potential incentive for development of products that generate more added value. - Too low price in relation to value could discourage development of new MPs in the long run; manufacturers are not rewarded for their innovation. - Opportunities for manufacturers for ‘gaming’ related to cost-effectiveness threshold and choice of comparator   *DE:*   - MP prices are more closely aligned with clinical benefits than before AMNOG.   *SE:*   - Inconsistent implementation of TLV decisions lead to differences in access to medications. | **Acceptability:**  *General:*   - Logical and fair *policy* to promote access and reward useful innovation.   *UK*:   - WTP-corresponding price levels may lead to uneven distributions of a product’s benefit surplus between payers and manufacturers, and volatility of R&D returns.   *DE:*   - Early public opinion supportive of AMNOG.   *FR:*   - Reimbursement being conditional on added therapeutic benefit over existing products priced at same or lower amount: manufacturers accept lower prices.   **Resource use:**  *General:*   - Evidence-based MP pricing supporting likewise MP access allows pharmacists to focus more on clinical and less on economic activities; - Value assessments are resource- and time-intensive.   **Feasibility:**  *General:*   - Difficult to implement, especially in therapeutic areas with no alternative treatment and patients suffering from severe life-threatening/debilitating disease; - Perceptions of value may differ across stakeholder groups;   *FR:*   - Price regulation is insufficient to control spending and should be combined with spending budgets.   *US:*   - Effective implementation of AMNOG-like system would require revisit of coverage and price negotiation rules. |
| Other policy interventions | [101, 102, 122, 123, 130, 131] | **Affordability:**  *US:*   - 340B Pricing Programme: reduced acquisition costs for oncology MPs.   **Availability:**  *US:*   - 340B Pricing Programme: reduced acquisition costs helped promote increase in access to high-cost oncology services.   **Sustainability:**  *JP:*   - Price Maintenance Premium: Potentially higher expenses for governments already concerned with high costs if premiums are overly generous.   **Equity:**  *US:*   - 340B Pricing Programme: evidence suggests increased access to oncology services in rural communities.   **Other impacts:**  *JP:*   - Price Maintenance Premium: Promotion of innovation. - Sales-related price revisions: penalisation of manufacturers, may discourage innovation.   *US:*   - 340B Pricing Programme: lack of specificity in patient eligibility guidelines enables too broad/narrow interpretation. | **Acceptability:**  *JP:*   - PMP can be considered a reasonable approach to evaluate value of new MPs.   **Resource use:** NA  **Feasibility:**  *JP:*   - MP price calculation generally expected to be difficult; actual market prices as basis would make it easy to calculate MP prices when incorporating market principles. |

^1^.Types of impacts of pricing policies are grouped into affordability, availability, equity, sustainability, and other.
^2^ Advantages/disadvantages of pricing policies are grouped into acceptability, resource use, and feasibility.

Abbreviations: AMNOG, Arzneimittelmarktneuordnungsgesetz (German Medicines Market Reorganisation Act); CA$, Canadian Dollar; EPR, external reference pricing; EU, European Union; GDP, gross domestic product; HTA, health technology assessment; IPR, internal reference pricing; LIC, low-income country; MP, medicinal product; MS, member state(s); NA, not available / not applicable; NICE, National Institute for Health and Care Excellence (England & Wales); OMP, medicinal product with an orphan designation; OOP, out of pocket; PDL, positive drugs list (Bulgaria); PMP, Price Maintenance Premium (Japan); R&D, research and development; TAU, temporary authorization for use (France); TLV, Tandvårds- och läkemedelsförmånsverket (Sweden); US$, United States Dollar; VIP, value-informed pricing, WTP, willingness to pay.
Country abbreviations: BE, Belgium; BG, Bulgaria; CA, Canada; CH, Switzerland; CO, Colombia; DE, Germany; EE, Estonia; ES, Spain; FR, France; GR, Greece; HR, Croatia; HU, Hungary; IE, Ireland; IT, Italy; JP, Japan; KR, South Korea; LV: Latvia; MX, Mexico; NL, Netherlands; NZ, New Zealand; PT, Portugal; RO, Romania; SE, Sweden; SK, Slovakia; TR, Türkiye; UK, United Kingdom; US, United States.
