# Supplementary material for: Price determinants and pricing policies concerning potentially innovative health technologies: a scoping review
Source: Eur J Health Econ. 2025 Sep 6;27(2):479–508. doi: 10.1007/s10198-025-01834-y (PMC13046678; doi:10.1007/s10198-025-01834-y)
Supplement: Supplementary file 5 — Supplementary file5 (DOCX 50 KB) [file 10198_2025_1834_MOESM5_ESM.docx]

# Online Resource 5: Access-related impact and organisational (dis-)advantages of applied pricing implementation methods in EEA/OECD member states – detailed overview

Table S5-1: Sources of evidence on access-related impact and organisational (dis-)advantages of applied price implementation methods (full overview)

| **Implementation method** | **References** | **Description of impact^1^** | **Description of (dis-)advantages^2^** |
| --- | --- | --- | --- |
| Price negotiations | [44, 45, 56, 57, 66, 73, 97, 116, 121, 132–135] | **Affordability:**  *General:*   - Policy sets limits on what purchasers pay for an MP.   *MX:*   - Prices of MPs on national formulary can be kept low due to monopsony and bargaining powers in negotiations. - However: Insufficient evidence for effectiveness of negotiation policies.   *US:*   - Medicare negotiations: Negotiations of MP prices with Medicare/Medicaid may improve MP affordability.   **Availability:**  *General:*   - Price negotiation after market entry should not delay patient access. - Availability depends on negotiation/arbitration outcomes and subsequent manufacturer decision; 29/148 MPs withdrawn from market following negotiation/arbitration (2011–2017); no clinically important medications affected from withdrawal. - Negotiation with monopsonistic purchaser leads to production of lower quantities at lower price compared to competitive market, impairing access to goods and services.   *MX:*   - Insufficient evidence for effectiveness of negotiation policies.   **Sustainability:**  *CA:*   - Prices negotiated through pCPA-led negotiations may lower MP expenditures.   *US:*   - Medicare negotiations: Price negotiation program to potentially save US$98.5 billion over 10 years (US$16.0 billion annually from 2029 onwards across Medicare Part B and D; initial estimate: US$288 billion); reduction of Medicare spending on Part B and D programmes by 5.4%.   **Equity**: NA  **Other impacts:**  *General:*   - Manufacturers risk losing an entire national market unless they negotiate.   *KR:*   - MP price negotiations confirmed need for improvements in transparency and consistency.   *US:*   - Medicare negotiations: Due to negotiation programme, manufacturers might increase US list prices; Pharmacy Benefit Manager revenue potentially impacted. - Medicare negotiations: manufacturers may attempt to evade price negotiation and inflation-linked penalties by introducing alternative versions of existing products. | **Acceptability:**  *DE:*   - Arbitration constitutes politically legitimate means for price setting in absence of negotiated agreement. - Price negotiations satisfactory for manufacturers, considering ongoing availability of novel MPs.   **Resource use:** NA  **Feasibility:** NA |
| Multi-indication pricing | [26, 32, 41, 111, 136–138, 141] | **Affordability:**  *General:*   - Potential decrease on payers’ expenditure; however, minimal impact or cost increase possible (existing/increased price in high-value indications could be justified). - Lower price linked to clinical value could alleviate pressure of limiting the use of high-priced MPs in broad patient populations.   *DE:*   - With each new indication, MPs’ list prices were significantly reduced.   *FR:*   - With each new indication, MPs’ list prices were significantly reduced.   **Availability:**  *General:*   - MPs may not be launched for certain low-value indications, even in countries applying weighted-average pricing or differential discounts.   *SE:*   - Fixed price across indications carries risk of suboptimal MP allocation where cost-effective indications are not reimbursed (and therefore not available).   **Sustainability:**  *General:*   - Potential net increase in spending due to access to MP that would otherwise not be paid at ‘asking price’.   **Equity:**  *General:*   - Patient with higher-value indication should not face the same cost sharing as patients receiving low value indication MP; administrative burden makes this alignment more difficult.   **Other impacts:**  *General:*   - Little economic incentive for manufacturers to launch indications in regions with smaller patient populations; launch would have adverse effect on list prices and thus on profits. - Might help align individual product access. - Might contribute to better alignment of MPs’ value and price. - Might optimise R&D incentives and increase competition. - Might increase transparency in MP use. - Might incentivise richer data systems. - Provides incentives to develop MP for high-value secondary indications. - Payers might become more reluctant to acknowledge added clinical value. - Manufacturer can capture all the economic surplus for each indication.   *IT:*   - Differences in (net) prices across indications (variation due to MEAs) do not necessarily represent differences in value.   *UK:*   - Increase in static and dynamic efficiency: prices will be set at cost-effective level, allowing access to more patients. | **Acceptability:**  *General:*   - Key stakeholders might oppose indication-specific pricing. - Different co-payments for the same MP depending on conditions may raise questions about equity of formulary design that will be difficult to answer in an understandable way.   *DE:*   - Pricing system embeds different value of a MP across indications/patient subgroups.   *ES:*   - Most stakeholders prefer ATC-based pricing to perceived implementation complexity and potential error associated with multi-indication MP pricing.   *UK:*   - Little use of flexible pricing scheme due to administrative complexity. - Large transactional burden associated with negotiating prices for each indication; simplicity is prioritised by payers.   **Resource use:**  *General:*   - Increase in administrative costs associated with identification of indications, differentiation of value, purchasing and payment process. - Data systems to administer models of indication-specific pricing may be complex and difficult to use and develop (concerns of stakeholders about inadequate data capabilities); uncertainty about resources that need to be devoted to successful implementation.   **Feasibility:**  *General:*   - Potential to interact with other pricing policies. - Requires adequate organisational infrastructure to obtain and record necessary clinical information, as well as underlying VIP system to determine value of MP and suitable institutional framework for evaluation of MPs. - Ex-ante estimation/ex-post tracking of use required per indication for weighted-average pricing/differential pricing. - Weighted-average pricing is complicated (considering competition and its impact on other MPs’ sales volume). - Legal/regulatory constraints might hinder application of multiple prices. - Privacy concerns. - Weighted-average pricing or value-based price for individual indications would reflect value of follow-on indications.   *DE:*   - Pricing system embeds different value of an MP across indications/patient subgroups.   *SE:*   - Weighted-average pricing: Assumes easy quantification of differences in therapeutic benefit.   *US:*   - Multiple brands for similar indications may be too burdensome or contribute to unnecessary confusion. |
| Price transparency | [55, 145, 147, 148, 150] | **Affordability:**  *CH:*   - Sharing negotiated prices of oncological MPs was associated with lower prices over time.   *New York/US:*   - Hospital Price Transparency: having price information may enable patients to be more in control and lower the costs they face from a medical situation.   *US:*   - Medicare/Medicaid Transparency Rule: May reduce OOP expenses for patients. - Access of hospitals to information on prices paid by other hospitals led to savings on brands for which they had previously paid relatively high prices. - Sharing prescription expenditure data among payers did not affect state Medicaid spending.   **Availability:** NA  **Sustainability:**  *General:*   - Inconclusive evidence whether MP pricing transparency reduces MP spending due to confidential agreements, nondisclosure of rebates/discounts, improper price reporting.   **Equity:** NA  **Other impacts:**  *General:*   - Manufacturers: confidential negotiations allow larger discounts to certain insurers to improve payers’ ability to negotiate lower prices.   *DE:*   - Possible positive collateral effects of net price disclosure: other countries can reference net prices on basis of an MP’s clinical value (more realistic than list prices). | **Acceptability:** NA  **Resource use:** NA  **Feasibility:**  *General:*   - Transparency alone is insufficient to encourage patients to price-shop; evidence suggests only modest changes in patient behaviour. - Combining transparency with targeted consumer incentives can lead to widespread price-shopping. - In combination with tangible financial incentive, MP spending can be reduced. |

^1^.Types of impacts of pricing policies are grouped into affordability, availability, equity, sustainability, and other.
^2^ Advantages/disadvantages of pricing policies are grouped into acceptability, resource use, and feasibility.

Abbreviations: ATC, anatomical therapeutic chemical; MEA, managed entry agreement; MP, medicinal product; NA, not available / not applicable; OOP, out of pocket; pCPA, Pan-Canadian Pharmaceutical Alliance; R&D, research and development; US$, United States Dollar, VIP, value-informed pricing.
Country abbreviations: CA, Canada; CH, Switzerland; DE, Germany; ES, Spain; FR, France; IT, Italy; KR, South Korea; MX, Mexico; SE, Sweden; SK, Slovakia; UK, United Kingdom; US, United States.
